# Estimation in discrete time coarsened multivariate longitudinal models

**DOI:** 10.1177/09622802231155010

**Published:** 2023-02-12

**Authors:** Marcus Westerberg

**Affiliations:** Department of Mathematics and Department of Surgical Sciences, Uppsala University, Regional Cancer Center Midsweden, Uppsala University Hospital, Uppsala, Sweden

**Keywords:** Coarsened data, longitudinal data, maximum likelihood, missing data, Monte Carlo expectation maximization, multi-state modeling

## Abstract

We consider the analysis of longitudinal data of multiple types of events where some of the events are observed on a coarser level (e.g. grouped) at some time points during the follow-up, for example, when certain events, such as disease progression, are only observable during parts of follow-up for some subjects, causing gaps in the data, or when the time of death is observed but the cause of death is unknown. In this case, there is missing data in key characteristics of the event history such as onset, time in state, and number of events. We derive the likelihood function, score and observed information under independent and non-informative coarsening, and conduct a simulation study where we compare bias, empirical standard errors, and confidence interval coverage of estimators based on direct maximum likelihood, Monte Carlo Expectation Maximisation, ignoring the coarsening thus acting as if no event occurred, and artificial right censoring at the first time of coarsening. Longitudinal data on drug prescriptions and survival in men receiving palliative treatment for prostate cancer is used to estimate the parameters of one of the data-generating models. We demonstrate that the performance depends on several factors, including sample size and type of coarsening.

## 1 Introduction

This article considers estimation of parameters of a discrete time multivariate longitudinal model, such as a competing risk survival model, an illness-death model, a progressive three-state model with an intermediate transient state, or a more general multi-state model,^1,2^ where data on some events are occasionally observed only on a coarser level than required. In this case, it may only be known that an event in a particular group of events has occurred during certain parts of the follow-up, for example, because some events are not recorded, resulting in gaps in the event history. This can occur by design, when subjects fail to self-report, or when data on events is registered in different linked data registers that cover different calendar periods. For example, data on drug prescriptions in Sweden is not available prior to the initiation of the Swedish Prescribed Drug Register in 2005, and consequently the start and duration of treatment(s) may be unknown for some study subjects. Historical data can be lost because it is not allowed to be stored indefinitely according to privacy and security laws (e.g. the General Data Protection Regulation [GDPR]) or because old hardware is upgraded (e.g. floppy disks to hard disk drives) without copying the old data to the new hardware. A register’s variable definitions may be updated, allowing a higher level of details to be recorded, and some events may only be distinguished from each other after this update. For example, the type of radiotherapy may only be known after one begins to register whether radiotherapy was curative or palliative. If there is an administrative lag of the assessment and registration of the cause of death, then the cause is not available for those dying during the latest calendar period.

When certain events are indistinguishable or not observable, we say that the data on events is coarsened. The concept of coarsened data is more general than missing data and describes several incomplete-data problems such as heaped and censored data.^3,4^ Multi-state models under different censoring mechanisms have been extensively studied both in continuous time^5–7^ and in discrete time.^8–12^ A special case of coarsening of counting process models in continuous time is the notion of a filtered counting process where no events can be observed during parts of the follow-up.^6^ Data coarsening can also occur on the time-scale due to the discretization of time into intervals.^13–15^ Whenever past events may affect the probability of future events, the coarsening of data on events simultaneously introduces missing data on events and time-dependent covariates defined by the history of the process. This is problematic since missing data may introduce bias and loss of efficiency unless appropriately dealt with.^16,17^ One approach to parameter estimation in continuous time models of recurrent events with gaps is to use a hot-deck imputation procedure that samples information from subjects with completely recorded history.^18,19^ To our knowledge, there are no previous studies that explicitly consider parameter estimation in more general coarsened discrete-time longitudinal models.

The aims of this article are to (1) introduce a representation of coarsened multivariate longitudinal data, (2) discuss estimators of the parameters of a general class of discrete-time coarsened longitudinal models, (3) study the performance of the estimators in a series of simulation studies, and (4) provide code for transparency and reproducibility. In particular, we focus on a coarsening process that, at each time, either coarsens the events into disjoint groups of events where it is only known if an event in a group occurred or not, or that right-censors the entire counting process. We derive the likelihood function under the assumption that the coarsening process is independent of the longitudinal process possibly given an observed external covariate process. We then restrict attention to models where the event probabilities are linked to linear predictors through a baseline category model and derive the score and observed information. The simulation studies involve three data-generating models of progressive type with either three or four states, where the coarsening generates gaps in the event history in different ways. Estimators based on full data maximum likelihood, the Monte Carlo Expectation Maximization (MCEM) algorithm,^20,21^ ignoring the coarsening and consequently acting as if no event occurred, and artificially right censoring at the first time of coarsening,^19^ are compared in terms of bias, empirical standard errors, and confidence interval coverage. For MCEM, importance sampling is used for approximating the expectations, and we show how to construct a proposal that generates nonzero weights for a certain class of models and coarsening.

## 2 The discrete-time longitudinal event process

In this section, we define the latent data $Y,T_{1}(Y),Z,H_{t},B_{t}$ for each subject in absence of coarsening, and derive the likelihood. This data is not fully observed when data is coarsened.

First, let τ<∞ be a known and fixed end of the follow-up of the study, and index time by integer values t∈{1,…,τ} . Index all possible event types (0,…,n) , with $n=n_{na}+n_{a}$ , where 0 is the reference event, $n_{na}\geq 0$ is the number of nonabsorbing events $\left(1,\ldots ,n_{na}\right)$ and $n_{a}\geq 0$ is the number of mutually exclusive absorbing (or terminal) events $\left(n_{na}+1,\ldots ,n\right)$ . In contrast to the continuous time case, event 0 is explicitly included and indicates that none of the other n events occurred, for example, that the subject stayed alive. For example, a man diagnosed with advanced prostate cancer may receive palliative treatment with a dose of Anti-Androgens (AA, event 1), or Gonadotropin-releasing hormone (GnRH, event 2), and may die because of prostate cancer (event 3), or other causes (event 4). Prior to any of events 1–4 and between any consecutive doses (events) of AA or GnRH, he experiences event 0.

Let $Y\;:\;=(Y_{1},\ldots ,Y_{\tau })$ be a (1+n)×τ matrix with column vectors $Y_{t}$ that has components $Y_{\;j,t}$ equal to 1 if event j occurred at time t and else equal to 0, called *latent local responses*.^9^ We assume that exactly one component of $Y_{t}$ jumps at each time, so $\sum_{\;j=0}^{n}Y_{\;j,t}=1$ , unless an absorption occurred at time t in which case all $Y_{\;j,s}=0$ for s>t . From hereon, we use analogous definitions implicitly for all other processes defined below, and let lowercase letters indicate realizations, so $y_{t}$ is a realization of $Y_{t}$ .

When an absorption occurs during the follow-up, let $T_{1}(Y)\;:\;=\min(1\leq t\leq \tau \;:\;\sum_{\;j=n_{na}+1}^{n}Y_{\;j,t}=1)$ be the time of absorption, and else set $T_{1}(Y)=\tau +1$ . Let $Z\;:\;=(Z_{1},\ldots ,Z_{\tau })$ be a q×τ matrix with column vectors $Z_{t}$ containing possibly time-dependent covariates and any time-independent covariates known at the start of follow-up. We restrict our attention to either the case that Z is time independent, that is, known at the start, or external, which implies that $Z_{t}$ is conditionally independent of any $Y_{s}$ for s<t given the history $Z_{1},\ldots ,Z_{t-1}$ .^9^ It is reasonable to assume that the covariate process is external whenever it is not generated by the subject itself (e.g. the amount of air pollution in a city). For notational convenience and brevity, we further restrict our attention to a discrete covariate process Z .

In addition to the above, we need to keep track of the history of $Y_{t}$ and $Z_{t}$ up until and including t , that is, $Y_{s}$ and $Z_{s}$ for all s≤t , which we denote by $H_{t}$ . It is also important to determine which events that can occur at time t given the history prior to t . We therefore let $B_{t}$ be a vector with components $B_{\;j,t}$ that are equal to 1 if the event j can occur at time t , that is, if the subject is at risk of event j , and else equal to 0. In the previous example, $B_{1,t}=0$ if a man has received GnRH prior to t and if it is not possible to receive AA (event 1) after having received GnRH. Therefore, the definition of $B_{t}$ as a function of the history is highly application-specific. Note that all $B_{\;j,t}$ are equal to 0 after an absorbing event, that $B_{t}$ is completely known at time t−1 given $H_{t-1}$ , and that it therefore defines the support of the conditional distribution of $Y_{t}$ given $H_{t-1}$ and $Z_{t}$ , in comparison with.^6^ Let θ be the parameter vector of interest and let $\theta \in \Theta \;:\;=R^{p}$ be the parameter space. Let ψ be a nuisance parameter vector, and assume that θ and ψ are separable (functionally independent). The conditional event probability of type j (discrete hazard)^6,12^ at time t depends on the parameter θ in the following way

(1) $$Pr_{\theta }(Y_{\;j,t}=1|H_{t-1}=h_{t-1},Z_{t}=z_{t})=\alpha_{\theta }(\;j,t|h_{t-1},z_{t})b_{\;j,t},$$

where the function $\alpha_{\theta }(\;j,t|h_{t},z_{t})$ is defined by the modeller. It satisfies $0<\alpha_{\theta }(\;j,t|h_{t},z_{t})<1$ for each j and t , and $\sum_{\;j=0}^{n}\alpha_{\theta }(\;j,t|h_{t},z_{t})b_{\;j,t}=1$ for each t up until and including the time of absorption.

Since Z is external, the joint probability mass function of (Y,Z) , denoted as $Pr_{\theta ,\psi }(\;y,z)$ , is proportional with respect to θ to

(2) $$\prod_{t=1}^{\tau }Pr_{\theta }(Y_{t}=y_{t}|H_{t-1}=h_{t-1},Z_{t}=z_{t}),$$

as shown in Appendix A of the Supplemental Material. Using (1), the above display is equal to what we call the *latent likelihood contribution*

(3) $$L^{latent}(\theta ;y,z)\;:\;=\prod_{t=1}^{\tau }\prod_{\;j=0}^{n}[\alpha_{\theta }(j,t|h_{t-1},z_{t})b_{\;j,t}{]}^{y_{\;j,t}}.$$

It depends on data that is latent (i.e. not directly observed) when data is coarsened.

## 3 The coarsening process and ignorable likelihood function

In this section, we define and describe how Y is coarsened by the coarsening process V and how the observed data $Y^{obs},Z^{obs},V^{obs},D,\Delta$ is produced. A summary of the introduced notation is provided in Supplemental Table 1.

Let V be a matrix of the same dimension as Y with corresponding column vectors $V_{t}$ and components $V_{\;j,t}$ equal to k if an event of type j is coarsened to group g at time t , with g∈{0,1,…,n} . If another event i also satisfies $V_{i,t}=g$ then events i and j are indistinguishable at time t , meaning that if event i or j occurred we only observe whether an event in group k occurred or not. For convenience, each group index is defined as the lowest event index in the group. We assume that absorbing events and nonabsorbing events are always coarsened to disjoint groups, unless the subject is right censored at time t , in which case all events are coarsened to group 0 for each time s≥t . This means that it is always known at each time whether there has been an absorbing event or if the process has been right censored. Note that if event j is the only event in its group then $V_{\;j,t}=j$ and event j is not coarsened. When $V_{\;j,t}=j$ for all j=0,…,n then there is no coarsening at time t .

The observed version of Y , called the *observed data local response* and denoted $Y^{obs}$ , is generated through $Y_{\;j,t}^{obs}\;:\;=\sum_{g=0}^{n}Y_{g,t}I(V_{g,t}=V_{\;j,t})$ . In particular, $Y_{\;j,t}^{obs}=1$ if an event in the group $V_{\;j,t}$ occurred at time t . Let $T_{2}(V)\;:\;=\min\{1\leq t\leq \tau \;:\;\sum_{\;j=0}^{n}V_{\;j,t}=0\}$ indicate the time of right censoring if it occurred, and $T_{2}(V)\;:\;=\tau +1$ if not. Let $D\;:\;=\min\{T_{1}(Y),T_{2}(V)\}$ indicate the first time of absorption or censoring and let $\Delta \;:\;=I(D=T_{2}(V))$ be an indicator of right censoring, where the indicator function I(A)=1 if A is true and else equals 0 . The last time of observed follow-up is D−Δ , so let $C_{t}\;:\;=I(D-\Delta \geq t)$ indicate that the subject has not yet experienced absorption and has not been right censored at time t .

V is only observed when the subject has not experienced absorption, so $V^{obs}$ has entries $V_{\;j,t}^{obs}\;:\;=C_{t}V_{\;j,t}$ and the observed covariate process has entries $Z_{\;j,t}^{obs}\;:\;=Z_{\;j,t}C_{t}$ . Note that $C_{t},D$ and Δ can be computed using $Y^{obs}$ and $V^{obs}$ and are therefore also observed, as explained in Appendix A of the Supplemental Material.

**Figure 1.**
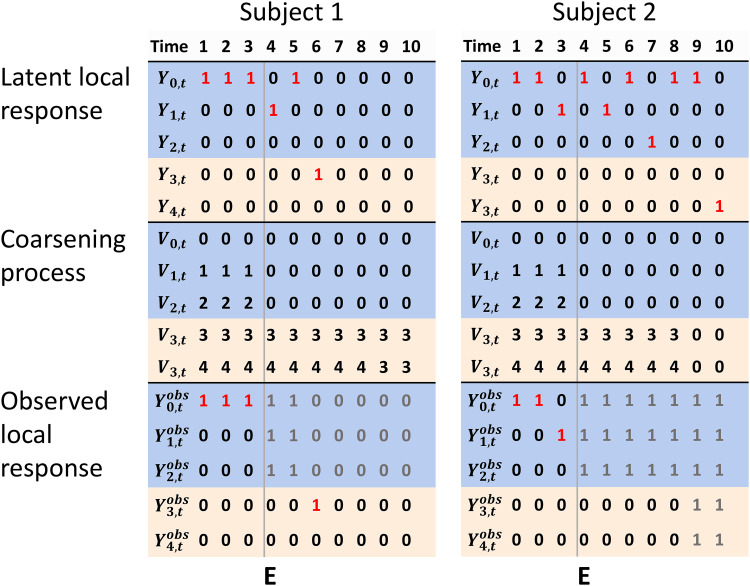
Examples of the latent local response, the coarsening process, and observed local response, for a coarsening process V defined by E —the time of start of coarsening of events 0, 1, and 2. Blue rows indicate event types 0, 1 (AA), and 2 (GnRH), while beige rows indicate the absorbing events 3 (death by prostate cancer) and 4 (death by other causes). Red 1’s indicate fully observed events. Gray numbers indicate that the corresponding events at those times were coarsened.

Continuing the previous example, we further assume that there are two registers, one that records drug prescriptions (events 1 and 2) and one that records date and cause of death (events 3 and 4) or date of emigration. Assume that the register that records drug prescriptions stops collecting information from time E=4 . Then V coarsens each event to its own group prior to time 4 , and it coarsens events 0 , 1 , and 2 , to group 0 , event 3 to group 3 and event 4 to group 4 from time t=4 and onward. Right censoring occurs at time $T_{2}(V)=9$ due to emigration, so from that time and onward it coarsens all events to group 0 . Formally, we have $V_{\;j,t}=jI(t<4)$ for j=0,…,2 and $V_{\;j,t}=jI(t<9)$ for j=4,5 . In Figure 1, we give examples of the latent local response, coarsening process and observed local response. Subject 1 received drug 1 at t=4 and died at t=6 because of prostate cancer. Subject 2 received drug 1 (AA) at t=3 and t=5 , and drug 2 (GnRH) at t=7 , and died at t=10 due to another cause. Gray numbers of the observed local response indicate the events that were coarsened to group 0. For subject 1, the drug received at t=4 was not observed, but had he died at t=4 then all events would have been observed despite the coarsening. For subject 2, it is unknown what types of drugs, if any, he received after t=4 .

**Figure 2.**
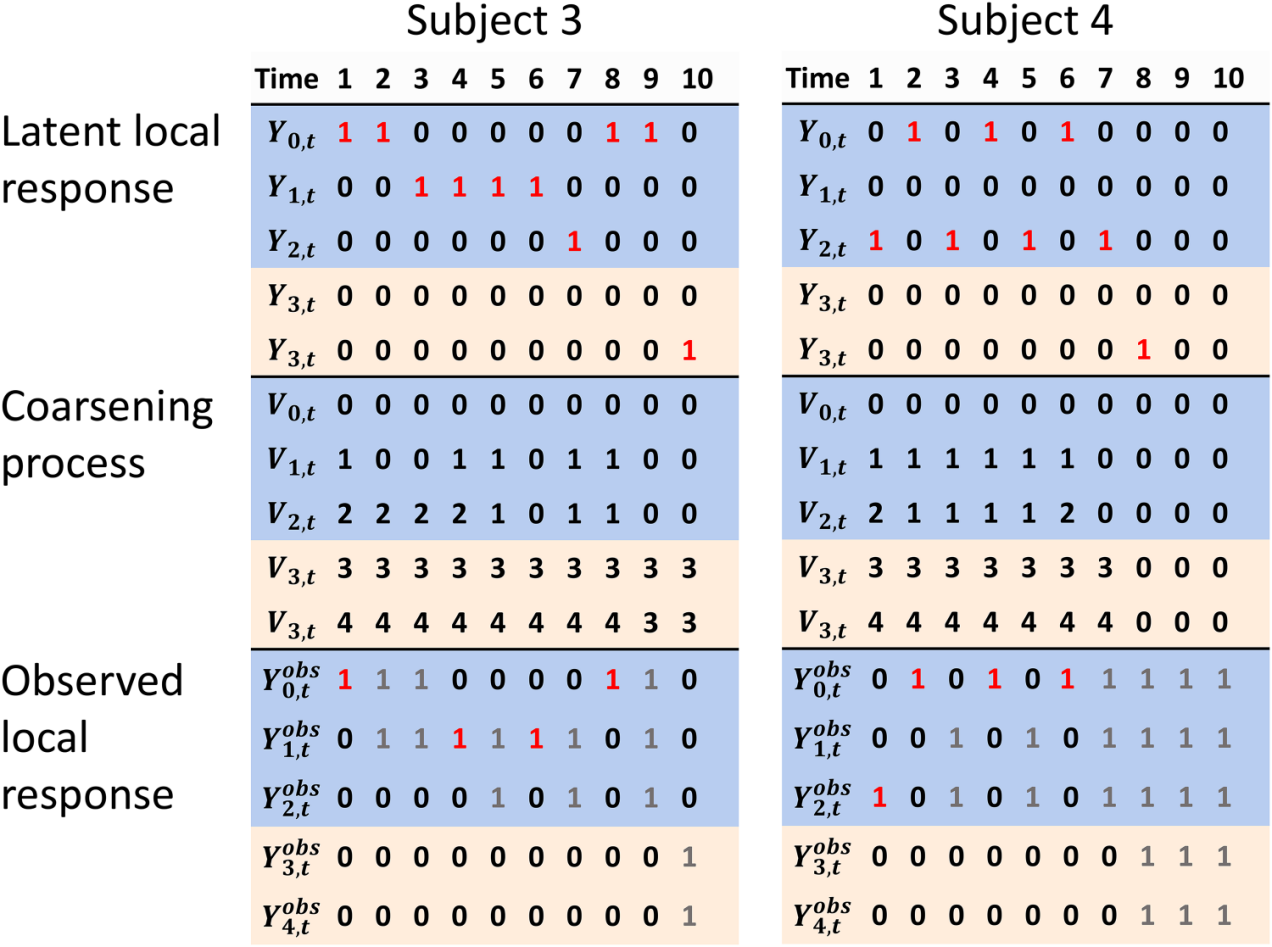
Examples of the latent local response, the coarsening process, and observed local response, for a randomly generated time-varying coarsening process V . Blue rows indicate event types 0, 1 (AA), and 2 (GnRH), while beige rows indicate the absorbing events 3 (death by prostate cancer) and 4 (death by other causes). Red 1’s indicate fully observed events. Gray numbers indicate that the corresponding events at those times were coarsened.

The groups that define the coarsening may vary over time (e.g. become larger and/or smaller) and subjects. In the previous example, this would correspond to a situation where the register that records drug prescriptions starts and stops collecting data at several different times. Figure 2 illustrates another situation with a different randomly generated time-varying coarsening process V . In this case, there are periods during which only partial information is collected. For example, it is not known whether event 0 or 1 (AA) occurred at times t=2 and 3 for subject 3 but it is known that he did not receive GnRH (event 2) at those times, and the cause of death is unknown.

It is important to stress that event though $Y_{t}$ is coarsened by $V_{t}$ it does not nimply that the event at time t has to be unknown, provided that enough information about the process before and after t is available. For example, assume that one cannot receive AA after having received GnRH. The two events 1 (AA) and 2 (GnRH) were coarsened to the same group at time t=5 for subject 3 in Figure 2, but event 1 was observed at t=6 , so under this assumption we know that event 1 must have occurred at t=5 . For the same reason, we know that subject 4 must have received GnRH at times 3 and 5.

### 3.1 The ignorable likelihood function

We derive the *ignorable likelihood function* that only depends on the observed data and θ . For notational convenience, we omit the dependency on the realizations v,y,z in the following. The joint probability mass function of V,Y,Z can be written as the product

(4) $$\prod_{t=1}^{\tau }Pr_{\theta ,\psi }(V_{t},Y_{t},Z_{t}|V_{\left[t-1\right]},Y_{\left[t-1\right]},Z_{\left[t-1\right]}).$$

We assume conditional independence between V and Y and that V is noninformative on θ such that each factor in (4) decomposes into two factors

(5) $$\prod_{t=1}^{\tau }Pr_{\theta ,\psi }(Y_{t},Z_{t}|Y_{\left[t-1\right]},Z_{\left[t-1\right]})\times Pr_{\psi }(V_{t}|V_{\left[t-1\right]},Z_{\left[t\right]}).$$

This assumption is reasonable whenever the events are observed by an external observer that does not affect the subject in any way by observing it, such as passive data registration of hospital visits or drug prescriptions in health-care registers, or when the observations occur at regular basis independently of the observed values, and when right censoring can be considered independent of Y given Z . When the coarsening is generated by the individual itself, for example due to failure to self-report, the validity of the conditional independence assumption relies on whether all covariates (such as disease severity and comorbidities) have been adequately measured or not and can be considered external or time-independent.

The joint probability mass function of the observed data is

(6) $$Pr_{\theta ,\psi }(Y^{obs}=y^{obs},Z^{obs}=z^{obs},V^{obs}=v^{obs}),$$

and in Appendix A of the Supplemental Material we show that (6) is proportional to what we define as the *ignorable likelihood contribution*, ignoring the coarsening

(7) $$L^{ign}(\theta ;y^{obs},z^{obs},v^{obs},d,\delta )\;:\;=\sum_{y_{\left[d-\delta \right]}\;:\;compatible}\prod_{t=1}^{d-\delta }\prod_{\;j=0}^{n}\{\alpha_{\theta }(\;j,t|h_{t-1},z_{t})b_{\;j,t}{\}}^{y_{\;j,t}}.$$

In the above display, *compatible* means that the hypothetical latent local response $y_{\left[d-\delta \right]}$ must agree with $y_{\left[d-\delta \right]}^{obs}$ , $v_{\left[d-\delta \right]}^{obs}$ , and the underlying model. This means in particular that $y_{\left[d-\delta \right]}$ must indicate events that could have occurred. In the previous example, it must indicate either event 0 or 1 at time 2 for subject 3 in Figure 2, and it can only indicate a sequence of events that can occur, so it can never indicate multiple deaths or a prescription of AA after GnRH.

The likelihood contribution is in other words the latent likelihood (3) evaluated up until the last time of follow-up d−δ and summed over all possible realizations y up to d−δ that are compatible with the observed data and the model. This is formally derived in Appendix A in the Supplemental Material (6). We denote this latent likelihood by

(8) $$L^{latent}(\theta ;y,z,d,\delta )\;:\;=\prod_{t=1}^{d-\delta }\prod_{\;j=0}^{n}\{\alpha_{\theta }(\;j,t|h_{t-1},z_{t})b_{\;j,t}{\}}^{y_{\;j,t}}.$$

The above derivation is applicable to any form of coarsening V that acts directly on the event process Y satisfying the conditional independence assumption. It does not encompass left-censoring since that introduces additional information about the past that is described by the underlying observed counting process and not by the observed data local response,^6^ and is out of scope for the current paper. However, the current setup may still allow (indirectly) for something that resembles left-censoring. For example, if one can only receive GnRH after having received at least one dose of AA, and we observe a GnRH prescription at time t but no prescriptions of AA (event 1) prior to t , then we know that event 1 must have occurred at least once at some time prior to t but we do not know when.

## 4 Maximum likelihood estimation

An estimate of θ can be obtained by maximizing the ignorable likelihood. The score and Hessian of the logarithm of the likelihood function are required in the numerical maximization of the likelihood function and used to construct confidence intervals. Details are provided in Appendix A of the Supplemental Material for the following type of longitudinal model and coarsening process.

We assume that the reference event 0 always can occur whenever the subject has not been absorbed with positive probability given the past, for example when it indicates that the subject stayed alive. We restrict our attention to the conditional (on the past) baseline category model.^9^ For t , there are n linear predictors $\eta_{\;j,t}(\theta )$ , one for each event (except 0, which is the baseline category). These are obtained by summing the model parameters relevant for the corresponding event multiplied with the corresponding (time-dependent) covariates specified by the user. These may include time itself, $z_{t}$ and summary measures of the history $h_{t-1}$ . The linear predictors define the discrete hazards through $\alpha_{\theta }(\;j,t|h_{t-1},z_{t})=\frac{e^{\eta_{\;j,t}(\theta )}}{1+\sum_{k=1}^{K}b_{k,t}e^{\eta_{k,t}(\theta )}}$ . This is the type of model that we later consider in the simulation studies.

### 4.1 Maximum likelihood using Monte Carlo Expectation Maximization (MCEM)

Direct maximization of the ignorable likelihood involves a large number of sums and products on the form (7) to be computed, which can become computationally intensive, for example, when the sample size N , end of follow-up τ and/or number of possible event types n are large and when coarsening is frequent and coarsens many events in few groups. As an alternative, we therefore consider the MCEM algorithm^20,22^ which is based on the original EM algorithm.^23^ Let $Y^{obs}$ be all the observed data for all subjects and let the corresponding latent data be Y . Let superscript k indicate subject k , with data $y^{k},z^{k},d^{k}$ and $\delta^{k}$ , and define the joint likelihood of the latent data of a sample of size N as

(9) $$f(y|\theta )\;:\;=\prod_{k=1}^{N}L^{latent}(\theta ;y^{k},z^{k},d^{k},\delta^{k}).$$

Let $\theta^{r}$ be a sequence of parameter values for $r=0,\ldots ,r_{max}$ , with $\theta^{0}$ being an initial value and $r_{max}$ being the maximum number of iterations specified by the user. The surrogate function $Q(\theta |\theta^{r})$ is defined as

(10) $$Q(\theta |\theta^{r})=E_{\theta^{r}}\left[\log\;f(Y|\theta )|Y^{obs}=y^{obs}\right].$$

The EM algorithm operates by iteratively computing the expectation $Q(\theta |\theta^{r})$ and then maximizing it with respect to θ , given a current estimate $\theta^{r}$ . The expectation in (10) is in general difficult to compute since the probability mass function of $Y_{\;j,t}^{k}$ for $t<d^{k}-\delta^{k}$ has to be obtained conditional on the observed data both before and after each time t . Also, $\alpha_{\theta }$ is in general a nonlinear function of the data and of θ . We therefore approximate (10) by use of importance sampling which we describe in the following section.

### 4.2 Importance sampling in MCEM

Importance sampling is a procedure to approximate a (conditional) expectation.^21,24^ It involves two probability mass functions (pmfs). For each individual with some coarsened data, one samples the latent data y up until time d−δ given the observed data. Ideally, these samples should come from the desired target pmf that is equal to the conditional pmf of the latent data given the observed data. In our case, however, it not easily sampled from. Instead, the samples are generated from a known proposal pmf from which it is relatively easy to produce samples. Importance weights are then used to compensate for the discrepancy between the proposal pmf and target pmf.

In our case, the target pmf is proportional to $L^{latent}(\theta ;y,z,d,\delta )$ which is the unconditional probability of the latent data and is easy to compute, but the computation of the normalization constant involves computing the corresponding quantity for all possible realizations given the observed data and may therefore be difficult to compute. Given a proposal pmf $q_{\theta }(y)$ and m independent Monte Carlo samples $y_{1},\ldots ,y_{m}$ from $q_{\theta }(y)$ , conditional expectations given the observed data can be approximated by weighted averages over the Monte Carlo samples, where the weights are normalized to avoid the exact computation of the normalization constant.^25,21^ In particular, the surrogate function $Q(\theta |\theta^{r})$ is approximated by $\sum_{k=1}^{N}\sum_{\;j=1}^{m}{\bar{w}}_{k,j}\log\;L^{latent}(\theta ;y^{k,j},z^{k},d^{k},\delta^{k})$ where the weights $w_{k,j}$ are defined as the ratio of the target and proposal pmfs evaluated at the Monte Carlo sample $y^{k,j}$ and the normalized weights are ${\tilde{w}}_{k,j}=w_{k,j}/\sum_{i=1}^{m}w_{k,i}$ . The support (set of realizations with nonzero probability) of the proposal pmf must contain the support of the target pmf, and ideally, they should match to avoid samples from the proposal pmf with zero weight.

### 4.3 Model restrictions

From hereon, we consider a relatively general type of longitudinal model and coarsening process for which it is computationally cheap to find the support of the target pmf and straightforward to construct a proposal pmf whose support matches the target pmf. In short, the support of $Y_{t}$ may only shrink over time, meaning that no event j with $B_{\;j,t}=0$ at some time t can have $B_{\;j,s}=1$ at some later time s>t . The coarsening process V may only coarsen nonabsorbing events with the reference event. Particular instances of this setup are studied in Section 5. Details for how to construct the proposal in this setting can be found Appendix B of the Supplemental Material.

For each subject, sampling is performed sequentially from t=1,…,d−δ and at each time t a realization $y_{t}$ is generated (which defines a realization of the history $h_{t}$ ). Let $P_{t}$ be a column vector with entries $P_{\;j,t}\;:\;=Pr(Y_{\;j,t}=1|h_{t-1},z_{t}^{obs})$ , then the target pmf is proportional to $\prod_{t=1}^{d-\delta }P_{t}^{T}y_{t}$ . Let J be a matrix of the same dimension as Y with columns $J_{t}$ and entries $J_{\;j,t}$ equal to 1 if event j can occur at time t given the observed data before and after t . Note that J is a function of observed data $y^{obs}$ and $v^{obs}$ . The conditional proposal pmf at time t given the past is defined as $Q_{t}\;:\;=\frac{J_{t}P_{t}}{J_{t}^{T}P_{t}}$ , and the proposal is the product $Q(y)=\prod_{t=1}^{d-\delta }Q_{t}^{T}y_{t}$ . The sampling algorithm is summarized in Algorithm 1.

**Algorithm 1.** Generating a sample and a weight.

**Require:** $J,\theta ,y^{obs},v^{obs},z^{obs},d,\delta$
initialize $y\leftarrow y^{obs}$
**for** t=1,…,d−δ **do**
compute $h_{t-1}$ from $y_{\left[t-1\right]},z_{\left[t-1\right]}^{obs}$
compute $P_{\;j,t}=Pr_{\theta }(Y_{\;j,t}=1|h_{t-1},z_{t}^{obs})$ , for j=0,…,n
compute $Q_{t}=\frac{J_{t}P_{t}}{J_{t}^{T}P_{t}}$
**if** $v_{0,t}^{obs}=0$ and $\sum_{\;j=0}^{n}y_{\;j,t}^{obs}=0$ **then**
sample ${\tilde{y}}_{t}∼Q_{t}$
set $y_{t}\leftarrow {\tilde{y}}_{t}$
**end if**
**end for**
compute $q_{\theta }(y)=\prod_{t=1}^{d-\delta }Q_{t}^{T}y_{t}$ and $p_{\theta }(y)=\prod_{t=1}^{d-\delta }P_{t}^{T}y_{t}$
compute $w=p_{\theta }(y)/q_{\theta }(y)$
**return** y,w

Further details regarding the MCEM implementation are provided in Appendix B in the Supplemental Material.

## 5 Simulation studies

**Figure 3.**
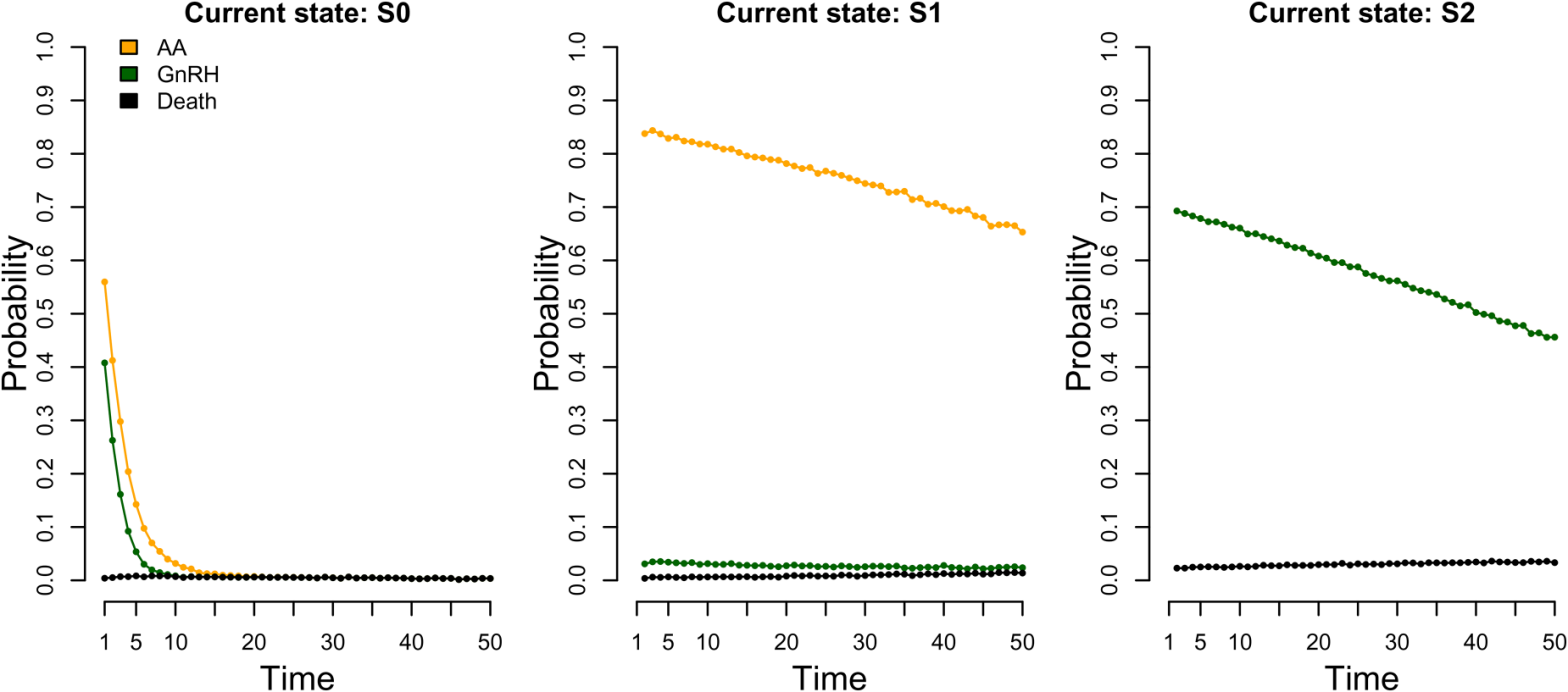
Approximate event probabilities (discrete time hazards) for data-generating model 1 at times 1,…,τ=50 , estimated via simulation of event histories of 100,000 subjects.

We considered three different data-generating models with different properties in a set of simulation studies. All models were based on the above specified discrete time conditional baseline category model. The first (model 1) concerned a study of men diagnosed with advanced prostate cancer assigned to palliative treatment with either AA or GnRH as in the examples in the previous sections. However, we only considered death from any cause instead of death from prostate cancer and other causes separately. During the follow-up a man could receive one or several doses of AA, and possibly switch to GnRH, or initiate GnRH treatment first without having received any AA. An initiation of a new drug, indicated by a prescription, was interpreted as a proxy for disease progression, and hence, in this study, a prescription of AA never followed a prior GnRH prescription. Therefore, the data-generating model included four events: reference 0 (Alive), 1 (AA), 2 (GnRH), and 3 (Death). The states were S0 (no prior treatment and alive), S1 (have received AA), S2 (have received GnRH). The discrete time hazards are shown in Figure 3 and indicate that most men initiated a treatment quite early on and frequently receive a dose of the same treatment.

The data-generating model parameters are shown in Supplemental Table 2. These were obtained by fitting the model on observed data on 16,312 men diagnosed with prostate cancer between 2006 and 2016, assigned to palliative treatment, and registered in Prostate Cancer data Base Sweden (PCBaSe).^26^ In short, PCBaSe contains linkages between the National Prostate Cancer Register of Sweden, the Prescribed Drug Registry, and the Cause of Death Registry. Follow-up extended to the first of the date of death, migration, or 2016-12-31. Data on dates of GnRH and AA prescriptions were obtained from the Prescribed Drug Registry, and the time was discretized using a 90-day time window. The study was approved by the Research Ethics Board in Uppsala (2016-239).

For model 1, we considered three scenarios with different follow-up and types of coarsening. A hypothetical blackout was simulated for some of the subjects independently of their event histories, during which no drug prescriptions were registered. In scenarios 1 and 2, prescriptions of GnRH were not registered, so events 0 and 2 were coarsened to the same group $\left(V_{0,t}=V_{2,t}=0\right)$ at such times t . Each subject was coarsened with probability 0.5 independently of the subject and of the other subjects. In scenario 1, the end of follow-up was τ=10 with coarsening at times t=3,4,5,6 . In scenario 2, τ=50 with coarsening at times t=5,6 and 20,…,29 . In scenario 3, τ=10 and coarsening occurred at times t=3,4,5,6 . In scenarios 1 and 2, there were only gaps in GnRH prescriptions, but in scenario 3 there were also gaps in prescriptions of AA such that the blackout acted on both GnRH and AA treatments independently. Thus, the coarsening process either grouped events 0 and 1, or 0 and 2, or all three nonabsorbing events, or neither. Each of the four patterns occurred with probability 0.25 .

Models 2 and 3 had three events: 0 (Alive), 1 (Treatment), and 2 (Death), and were designed to explore two other qualitatively different dynamics, see Supplemental Figures 1 and 2 and Supplemental Tables 3 and 4 for visualizations of the discrete hazards and model parameters. For model 2, follow-up ended at τ=50 and treatment initiation was infrequent, increased with time, and repeated treatments were also infrequent but decreased with time, while risk of death was distinctively different for treated and untreated subjects. For model 3, τ=10 , treatment initiation was infrequent, and risk of death increased rapidly towards t=10 , while additional treatment was frequent among already treated, especially during times 1–6. In both cases, gaps in the treatment history were generated with probability 0.5 . For model 2, this occurred at times t=1,…,10 , and immediately caused a large gap for a large portion of the study population, and for model 3 it occurred in the middle of the follow-up at times t=3,4,5,6 .

### 5.1 Estimators

The full data MLE (MLE*) was computed before coarsening the data. We considered four estimators used after coarsening. The first (EarlyCensor) is an MLE on a modified dataset obtained by artificially right censoring each subject at the first time t where $v_{\;j,t}^{obs}=0$ for some j>0 . We do not expect this to introduce any structural bias since the right censoring is conditionally independent of Y and non-informative whenever V is, but it leads to an unnecessary loss of information. The second (IgnoreV) was inspired by the *last observation carried forward* approach, constructed by artificially modifying the data by redefining $y^{obs}$ at times where $\sum_{\;j=0}^{n}y_{\;j,t}^{obs}>1$ to indicate event 0, and subsequently, estimate parameters using the MLE as if data was not coarsened. The third was full data maximum likelihood (DirectML) obtained by numerically summing over all realizations, as in (7).

For all the above estimators, the inverse of the negative Hessian of the corresponding log-likelihood at convergence was computed based on the (modified data) and its diagonal elements were used as estimates of the standard error and to construct 95% approximate confidence intervals under the assumption of asymptotic normality.^27^

The last estimator was based on MCEM and importance sampling, as described in the previous section. The parameter sequence $\theta^{r}$ was initialized using EarlyCensor. Then, Q was maximized iteratively based on sequentially generated Monte Carlo estimates of the expected gradient and Hessian of the latent likelihood. The procedure is summarized in Algorithm 2. To stabilize the sequence of parameter updates, a cumulative weighted average of parameter estimates obtained from the MCEM procedure was used. See Appendix B of the Supplemental Material for more details on how the MCEM algorithm was implemented. The number of Monte Carlo samples $m_{r}$ was initially set to $m_{1}=10$ and increased by 5 with each iteration until m=25 for all simulation studies except for model 1 scenario 2, where m=40 . See Table 5 in the Supplemental Material for a summary of these settings.

**Algorithm 2.** MCEM.

initialize $\theta^{0}$ using EarlyCensor
**for** r=1,…,10 **do**
$m_{r}\leftarrow \min(10+5(r-1),m)$
approximate $Q(\theta |\theta^{r-1})$ using $m_{r}$ Monte Carlo samples
obtain $\hat{\theta }$ by maximizing the approximation of $Q(\theta |\theta^{r-1})$
**if** r1 **then**
update $\theta^{r}\leftarrow (\hat{\theta }m_{r}+\sum_{\;j=1}^{r-1}{\hat{\theta }}^{\;j}m_{j})/\sum_{\;j=1}^{r}m_{j}$
**else**
update $\theta^{1}\leftarrow \hat{\theta }$
**end if**
**end for**
**return** $\theta^{r}$

We assessed bias, empirical standard error (EmpSE), and confidence interval coverage (%) for each parameter and estimator, along with corresponding Monte Carlo standard errors (MC-SE), as described in Morris et al.^28^

### 5.2 Simulation settings

For model 1, scenarios 1 and 3, the number of repetitions was set to $n_{sim}=$ 1024 for sample size N = 1000, and this choice was based on an initial small simulation run of scenario 1. In this case, the estimated standard deviation across the simulations of each component of the parameter vector θ in the preliminary run was far below 2. Thus, $n_{sim}=$ 1 024 was conservative since the MC-SE was $\sqrt{2/1024}<0.05$ .^28^ In scenario 2, $n_{sim}$ was set to 512 for N = 1000 which was limited by the significantly longer simulation time compared to scenario 1 due to the longer follow-up. At this settings, with all standard errors estimated to be smaller that 0.5 based on a preliminary run, each MC-SE of the bias was at most 0.05 which was considered satisfactory. Due to computational complexity and scale, DirectML was not used in scenario 2.

For models 2 and 3, similar reasoning was used to determine the settings for model 2 ( $n_{sim}=$ 512) and model 3 ( $n_{sim}=$ 1024). See Supplemental Table 5 for a summary of the simulation settings for all sample sizes. All computations were performed using R version 4.1.0,^29^ and we used the R function *nlm* for function minimization that is based on a Newton-type algorithm.

### 5.3 Results

The bias of all estimators decreased for each parameter with increasing sample size, except for IgnoreV for some parameters. Although all estimators were biased for one or many parameters at a sample size N=500 , most were approximately unbiased at N=10,000 (typically within two MC-SE of true parameter values). IgnoreV was biased for some or most parameters for each model and scenario, and the bias was negative for intercept terms and positive for time variables, except for model 3 where the opposite was observed for the event *Death* and variables *S0 intercept* and *S0 time*. EmpSE’s decreased with sample size and was smallest for MLE* followed by DirectML and MCEM and largest for EarlyCensor. Coverage of 95% confidence intervals was generally close to 95% for these estimators for all sample sizes and models, while coverage for IgnoreV was poor in most cases. All results are provided in Supplemental Tables 6 to 20.

For model 1, scenario 1, the biases of DirectML were similar to the MLE without coarsening (MLE*), while EarlyCensor had larger bias for some parameters at N=500 , Table 1 and Supplemental Figure 3. MCEM had close to identical bias, EmpSE and coverage compared to DirectML, although some bias remained for a few parameters at N=10000 , e.g. *GnRH, S1 intercept*. IgnoreV was clearly biased for several parameters, for example, *GnRH, S1 intercept*, and *GnRH, S1 time* and the coverage was close to 95% occasionally when bias was small (e.g. *Death, S0 intercept*).

**Table 1.** Model 1, scenario 1. Bias of each parameter by estimator. MLE* is the MLE obtained without coarsening.

		GnRH	GnRH	Death	Death
		S1 intercept	S1 time	S0 intercept	S0 time
Truth		− 1.62	− 0.039	− 4.66	− 0.023
estimator	*N*	Bias (MC-SE)	Bias (MC-SE)	Bias (MC-SE)	Bias (MC-SE)
MLE*	500	− 0.048 (0.018)	0.001 (0.003)	− 0.023 (0.038)	− 0.103 (0.027)
	1000	− 0.003 (0.012)	− 0.001 (0.002)	− 0.057 (0.015)	− 0.005 (0.003)
	10,000	− 0.008 (0.008)	0.001 (0.001)	0.007 (0.009)	− 0.001 (0.002)
IgnoreV	500	− 0.991 (0.020)	0.129 (0.003)	− 0.109 (0.026)	0.011 (0.013)
	1000	− 0.933 (0.013)	0.125 (0.002)	− 0.082 (0.013)	0.036 (0.003)
	10,000	− 0.928 (0.008)	0.125 (0.001)	− 0.023 (0.008)	0.036 (0.001)
EarlyCensor	500	− 0.096 (0.024)	0.004 (0.004)	0.178 (0.114)	− 0.434 (0.059)
	1000	− 0.013 (0.015)	− 0.001 (0.002)	− 0.030 (0.023)	− 0.045 (0.014)
	10,000	− 0.008 (0.009)	0.001 (0.001)	0.008 (0.009)	− 0.003 (0.002)
DirectML	500	− 0.052 (0.018)	0.001 (0.003)	0.050 (0.104)	− 0.254 (0.045)
	1000	− 0.003 (0.012)	− 0.001 (0.002)	− 0.035 (0.020)	− 0.027 (0.013)
	10,000	− 0.008 (0.008)	0.001 (0.001)	0.006 (0.009)	− 0.001 (0.002)
MCEM	500	− 0.090 (0.018)	0.007 (0.003)	0.073 (0.107)	− 0.289 (0.048)
	1000	− 0.037 (0.012)	0.004 (0.002)	− 0.035 (0.018)	− 0.023 (0.010)
	10,000	− 0.042 (0.008)	0.006 (0.001)	0.007 (0.009)	0.000 (0.002)

For model 1, scenario 2, the follow-up was longer ( τ=50 vs. τ=10 ) and the coarsening occurred later during the follow-up. The bias was clearly smaller for all parameters and all estimators, compared to scenario 1, Table 2 and Supplemental Figure 4. In particular, the biases of EarlyCensor and MCEM were comparable to MLE* at N= 10,000, and the bias of IgnoreV was smaller compared to scenario 1 (e.g. *GnRH, S2 had S1*) or almost negligable (e.g. *GnRH, S1 time*). The EmpSEs of the estimators were considerably smaller in scenario 2 compared to 1.

**Table 2.** Model 1, scenario 2. Bias (MC-SE) of each estimator. MLE* is the MLE obtained without coarsening.

		GnRH	GnRH	Death	Death
		S1 intercept	S1 time	S0 intercept	S0 time
Truth		− 1.62	− 0.039	− 4.66	− 0.023
estimator	*N*	Bias (MC-SE)	Bias (MC-SE)	Bias (MC-SE)	Bias (MC-SE)
MLE*	500	0.001 (0.010)	0.000 (0.000)	− 0.005 (0.018)	− 0.003 (0.001)
	1000	0.014 (0.007)	0.000 (0.000)	− 0.003 (0.013)	0.000 (0.001)
	10,000	0.002 (0.004)	0.000 (0.000)	− 0.007 (0.007)	0.000 (0.000)
IgnoreV	500	− 0.132 (0.010)	0.003 (0.000)	0.003 (0.018)	− 0.003 (0.001)
	1000	− 0.119 (0.007)	0.002 (0.000)	0.006 (0.013)	− 0.001 (0.001)
	10,000	− 0.129 (0.004)	0.003 (0.000)	0.002 (0.007)	0.000 (0.000)
EarlyCensor	500	− 0.011 (0.013)	0.000 (0.001)	− 0.009 (0.026)	− 0.019 (0.010)
	1000	0.007 (0.008)	0.000 (0.000)	− 0.003 (0.015)	− 0.002 (0.001)
	10,000	0.003 (0.005)	0.000 (0.000)	0.002 (0.008)	− 0.001 (0.000)
MCEM	500	− 0.005 (0.010)	0.000 (0.000)	− 0.008 (0.019)	− 0.003 (0.001)
	1000	0.009 (0.007)	0.000 (0.000)	− 0.003 (0.013)	0.000 (0.001)
	10,000	− 0.005 (0.004)	0.000 (0.000)	− 0.007 (0.007)	0.000 (0.000)

For model 1, scenario 3, both the events *GnRH* and *AA* were coarsened instead of only *GnRH* as in scenarios 1 and 2. Biases were significantly larger for IgnoreV and larger for EarlyCensor in scenario 3 compared to scenario 1, in particular for parameters *S1 intercept* and *S1 time* for all events *AA, GnRH, and Death*, Table 3 and Supplemental Figure 5. Coverage for IgnoreV was far below 95% for most parameters (except e.g. *Death, S0 intercept*). MCEM had larger biases and EmpSE’s for N = 500 and 1000 but had more comparable performance at N = 10,000, relative to scenario 1. There were in general larger differences in terms of EmpSEs between the estimators, favouring DirectML and MCEM and disfavouring EarlyCensor, compared to scenario 1.

**Table 3.** Model 1, scenario 3. Bias (MC-SE) of each parameter by estimator. MLE* is the MLE obtained without coarsening.

		GnRH	GnRH	Death	Death
		S1 intercept	S1 time	S0 intercept	S0 time
Truth		− 1.62	− 0.039	− 4.66	− 0.023
estimator	*N*	Bias (MC-SE)	Bias (MC-SE)	Bias (MC-SE)	Bias (MC-SE)
MLE*	500	− 0.044 (0.018)	0.003 (0.003)	− 0.033 (0.031)	− 0.067 (0.020)
	1000	0.001 (0.012)	− 0.003 (0.002)	− 0.032 (0.016)	− 0.013 (0.004)
	10,000	− 0.013 (0.009)	0.002 (0.001)	− 0.007 (0.012)	− 0.001 (0.002)
IgnoreV	500	− 2.393 (0.020)	0.265 (0.003)	− 0.135 (0.023)	0.021 (0.010)
	1000	− 2.335 (0.013)	0.258 (0.002)	− 0.103 (0.014)	0.036 (0.003)
	10,000	− 2.345 (0.009)	0.262 (0.001)	− 0.071 (0.010)	0.040 (0.002)
EarlyCensor	500	− 0.078 (0.035)	− 0.010 (0.010)	0.592 (0.127)	− 0.854 (0.082)
	1000	− 0.021 (0.019)	− 0.005 (0.003)	0.114 (0.097)	− 0.223 (0.038)
	10,000	− 0.016 (0.013)	0.001 (0.002)	− 0.018 (0.013)	0.002 (0.004)
DirectML	500	− 0.035 (0.019)	0.002 (0.003)	0.151 (0.050)	− 0.259 (0.042)
	1000	0.006 (0.013)	− 0.004 (0.002)	0.004 (0.020)	− 0.043 (0.012)
	10,000	− 0.013 (0.010)	0.002 (0.001)	− 0.004 (0.011)	− 0.002 (0.003)
MCEM	500	− 0.106 (0.020)	0.012 (0.003)	0.145 (0.106)	− 0.322 (0.052)
	1000	− 0.057 (0.013)	0.006 (0.002)	0.022 (0.021)	− 0.055 (0.014)
	10,000	− 0.077 (0.010)	0.011 (0.001)	− 0.003 (0.011)	− 0.001 (0.002)

In contrast to model 1, models 2 and 3 have three events instead of four, and these two models have different parameter values and follow-up ( τ=100 vs. τ=10 ). Transition to state S1 (*Treatment*) occurs late and early, respectively, and the probability of receiving additional treatment after this transition was low and high, correspondingly. For model 2, IgnoreV was close to unbiased for several parameters (e.g. *Treatment, S1 intercept* and *Treatment, S1 time*) and coverage was satisfactory in these cases. For model 3, IgnoreV was biased for the same corresponding parameters with poor coverage (especially for N = 10 000), Supplemental Figures 6 and 7. MCEM was slightly biased for some parameters, such as *Death, S1 intercept*), even at N = 10,000. MCEM also had smaller or comparable EmpSE’s compared to DirectML (e.g 0.550 vs. 0.563 for the same parameter at N = 1000), and coverage was somewhat poor, between 89 and 94.9% at N=10,000 .

## 6 Discussion

In this article, we derived the likelihood function for discrete time counting process models under independent and noninformative coarsening, and compared the performance of several estimators through a series of simulation studies, focusing on bias, empirical standard error, and 95% confidence interval coverage. Our article adds to the literature on discrete time counting process modelling by providing the appropriate set-up, expressions for the likelihood with a coarsening process, and expressions for derivatives including the score and observed information under the conditional baseline category model. We derived and implemented the direct maximum likelihood estimator, and showed how to construct a proposal distribution used in an importance sampling MCEM algorithm for certain models and coarsening.

We saw that the maximum likelihood estimation for discrete time counting process models in the absence of a coarsening process (MLE*) was biased in smaller samples but 95% CIs still reached approximately 95% coverage. The bias shrunk more slowly for DirectML, MCEM and EarlyCensor with increasing sample size, which is expected due to the loss of information due to data coarsening. Ignoring the coarsening (IgnoreV) introduced mild to strong bias in varying direction and magnitude, depending on the data-generating model and the timing and amount of coarsening, and confidence interval coverage was generally poor. For example, relative to scenario 1, the bias of IgnoreV increased when the coarsening became coarser in scenario 3. For model 2, where follow-up was long ( τ=50 ) and treatment initialization was infrequent during the first 10 time steps when coarsening could occur, bias of IgnoreV was relatively small for most parameters, and in contrast, larger for model 3 where treatment initiation was frequent early on and during times of coarsening. This behavior is expected since IgnoreV simultaneously introduced misclassification in the time-dependent covariates that summarize the history $H_{t}$ and in the outcome $Y_{t}$ , and this misclassification correlated over time when the coarsening occurred at multiple time points.^30^ The magnitude of the misclassifaction likely depended on the true discrete hazard of event 0, which was high for model 2 during times when data was coarsened.

Directly maximizing the likelihood (DirectML) gave the smallest empirical standard errors as expected (where it was used), often closely followed by MCEM, and these were occasionally significantly larger for EarlyCensor. This was not surprising since EarlyCensor disregards all information after first time of censoring while the other two estimators use all available information. In particular, both EarlyCensor and MCEM performed better in terms of bias and EmpSE for model 1 in scenario 2 compared to 1, which is expected since (a) event 2 (GnRH) most likely occurred before coarsening (prior to time 5) in scenario 2, (b) the follow-up was longer, and (c) the coarsening process acted later during the follow-up and at that time fewer men were alive.

An important limitation of the DirectML estimator is the need to not only compute all possible realizations that are compatible with the observed data, but also to propagate the history of events forward in time when computing each such realization. The number of computations required for the first operation scales exponentially with the number of time points where the event is unknown and polynomially in the number of possible events that could have occurred at each such time point. Although these computations only need to be performed once before optimization, each realization requires some computational time and storage. Thus, for large sample sizes where events are frequently coarsened into one of few large groups over a long time of follow-up, DirectML may quickly become computationally expensive.

In this regime, the limitations of DirectML can be accommodated using an instance of MCEM with importance sampling which is less computationally intensive. Whenever both can be used, we saw that it performed similarly to DirectML. In practice, the performance of MCEM can be improved further by increasing the number of Monte Carlo samples and iterations, for convergence guarantees to hold,^21^ to alleviate the occasional bias and poor coverage of MCEM that was observed. With some further optimization of code, use of convergence criteria, and automation of MCEM, for example, by dynamically increasing the number of MC samples at each iteration,^31^ we expect there to be many situations where MCEM is computationally feasible but where DirectML is not. Alternative approaches with potentially more optimal use of the computational budget include Markov Chain Monte Carlo methods and stochastic version of the EM algorithm.^22,32^

It could be attractive to increase the time-interval width when discretizing time in order to reduce computational complexity, but the use of large widths may lead to biased inferences.^13–15^ Future work should therefore instead investigate ways to reduce the computational cost using state of the art Monte Carlo methods in combination with the EM algorithm instead of importance sampling-based MCEM. We speculate that artificial right censoring (EarlyCensor) could be a viable option worth attention when sample size is very large, especially if coarsening occurs for the first time late during the follow-up relative to the model under consideration, and at many time points. In this regime, we expect that EarlyCensor may have negligible bias and the loss in precision to be moderate. However, the artificial censoring time was conditionally independent of Y since Y was conditionally independent of V , and EarlyCensor may not necessarily be asymptotically unbiased when there is some dependence between V and Y . Future work should therefore also consider modeling the dependence between Y and V and appropriate modifications of the proposed estimators. It also remains to prove consistency and asymptotic normality of the proposed estimators before and after coarsening under suitable regularity conditions.

In conclusion, we have proposed several useful estimators of model parameters in discrete time coarsened counting process models, with partially complementary areas of use. From our experience, coarsened data and missing event types are common problems encountered in studies based on data collected from multiple registers and inappropriate handling of such data can lead to biased inference as demonstrated.

## Supplemental Material

sj-pdf-1-smm-10.1177_09622802231155010 - Supplemental material for Estimation in discrete time coarsened multivariate longitudinal models Click here for additional data file. Supplemental material, sj-pdf-1-smm-10.1177_09622802231155010 for Estimation in discrete time coarsened multivariate longitudinal models by Marcus Westerberg in Statistical Methods in Medical Research

sj-zip-2-smm-10.1177_09622802231155010 - Supplemental material for Estimation in discrete time coarsened multivariate longitudinal models Click here for additional data file. Supplemental material, sj-zip-2-smm-10.1177_09622802231155010 for Estimation in discrete time coarsened multivariate longitudinal models by Marcus Westerberg in Statistical Methods in Medical Research

sj-zip-3-smm-10.1177_09622802231155010 - Supplemental material for Estimation in discrete time coarsened multivariate longitudinal models Click here for additional data file. Supplemental material, sj-zip-3-smm-10.1177_09622802231155010 for Estimation in discrete time coarsened multivariate longitudinal models by Marcus Westerberg in Statistical Methods in Medical Research

sj-zip-4-smm-10.1177_09622802231155010 - Supplemental material for Estimation in discrete time coarsened multivariate longitudinal models Click here for additional data file. Supplemental material, sj-zip-4-smm-10.1177_09622802231155010 for Estimation in discrete time coarsened multivariate longitudinal models by Marcus Westerberg in Statistical Methods in Medical Research
