# Supplementary material for: Estimation in discrete time coarsened multivariate longitudinal models
Source: Stat Methods Med Res. 2023 Feb 12;32(4):806–19. doi: 10.1177/09622802231155010 (PMC10119900; doi:10.1177/09622802231155010)
Supplement: sj-pdf-1-smm-10.1177_09622802231155010 - Supplemental material for Estimation in discrete time coarsened multivariate longitudinal models [file sj-pdf-1-smm-10.1177_09622802231155010.pdf]

Supplement to: Estimation in discrete time coarsened multivariate longitudinal models

Marcus Westerberg

Department of Mathematics and Department of Surgical Sciences

Uppsala University

marcus.westerberg@uu.se

January 16, 2023

1 Contents

This document contains three appendices. Appendix A provides a derivation of the joint probability mass function (pmf) of the observed data and the ignorable likelihood function, and provides all the partial derivatives of the logarithm of the ignorable likelihood function. Appendix B provides details on the implementation of the Monte Carlo Expectation Maximization (MCEM) algorithm. Additional details about the simulation study are found in Appendix C.

2 Appendix A: The log-likelihood function and its partial derivatives

2.1 Notation

A list of key notation introduced in this paper is provided in Supplementary Table 1 below. Recall in particular that D equals the first time of absorption or censoring, Δ is an indicator of right censoring, and $C_t := I(D-\Delta \geq t)$.

Let $\mathbb{Y}$ be the the sample space of Y that only includes realizations with nonzero probability, and let $\mathbb{V}$ and $\mathbb{Z}$ be the corresponding sample spaces for V and Z. The observed data Y^{obs} and Z^{obs}, and V^{obs} are represented using functions of the latent data Y and Z, and V using notations similar that used by [1] on coarsened data, but adapted to this situation where data coarsening is not monotone. For realizations of the latent data v, y, z we obtain the observed data as functions of the latent data

$$\begin{aligned} G^1_{v^{obs}}(y) &:= y^{obs}, \\ G^2_c(z) &:= z^{obs}, \\ G^3_c(v) &:= v^{obs}, \end{aligned} \tag{1}$$

where c, d, and δ are omitted since these follow from y^{obs} and v^{obs}. Let $[t]$ appended to a process indicate all data within the interval 1 to t, e.g. $Y_{[t]} = (Y_1, \ldots, Y_t)$, defined analogously for realizations and sample spaces, and define the functions in (1) analogously when applied on data between times 1 to t.

Supplementary Table 1: List of notation

Variable	Description
N	sample size
τ	administrative end of follow-up
n	number of possible event types (except the reference event)
n_a	number of absorbing events
$Y_{j,t}$	equal to 1 if event j occurred at time t, else 0
Y_t	column vector of all the local responses at time t
Y	matrix of all Y_t from $t = 1$ to τ
$T_1(Y)$	time of absorption, else $\tau + 1$
Z_t	vector of q covariates at time t
H_t	history of Y_t and Z_t up to time t
$B_{j,t}$	equal to 1 if event j could occur at time t, else 0
$V_{j,t}$	equal to k if event j is coarsened to group k at time t
V_t	column vector of all the $V_{j,t}$ at time t
V	a matrix of all V_t from $t = 1$ to τ
$T_2(V)$	time of right censoring if it occurred, else $\tau + 1$
$I(A)$	the indicator function of event A, equal to 1 if A occurred, else 0
$G^1_{v^{obs}}(y)$	equal to y^{obs}, the observed part of y
$G^2_c(z)$	equal to z^{obs}, the observed part of z
$G^3_c(v)$	equal to v^{obs}, the observed part of v

Note that C, D and Δ can be computed using Y^{obs} and V^{obs} and are therefore also observed. To see this, recall that $T_2(V^{obs})$ returns the first time t when all components of V_t^{obs} are zero, which is equal to the first time when C_t is zero, if this occurs. If not, then $T_2(V^{obs})$ is equal to $\tau + 1$ and $C_t = 1$ for all t. Thus, C is known, and therefore $D - \Delta$. Also note that an absorption occurred at the last time t where $C_t = 1$ only if $\sum_{j=n_{na}+1}^{n} Y_{j,t}^{obs} > 0$. This determines Δ and D.

2.2 The latent likelihood contribution

Since Z is external, the joint pmf of (Y, Z), denoted as $Pr_{\theta,\psi}(y, z)$, is proportional, with respect to θ, to a product of the conditional pmf of Y_t

$$\begin{aligned}
Pr_{\theta,\psi}(y, z) &= \prod_{t=1}^{\tau} Pr_{\theta,\psi}(Y_t = y_t, Z_t = z_t | H_{t-1} = h_{t-1}) \\
&= \prod_{t=1}^{\tau} Pr_{\theta}(Y_t = y_t | H_{t-1} = h_{t-1}, Z_t = z_t) \\
&\quad \times Pr_{\psi}(Z_t = z_t | H_{t-1}) \\
&\propto \prod_{t=1}^{\tau} Pr_{\theta}(Y_t = y_t | H_{t-1} = h_{t-1}, Z_t = z_t),
\end{aligned} \tag{2}$$

where $\propto$ denotes that the right is proportional to the left with respect to θ. The last row in the above display is equal to the *latent likelihood contribution*

$$L^{latent}(\theta; y, z) := \prod_{t=1}^{\tau} \prod_{j=0}^{n} [\alpha_\theta(j, t | h_{t-1}, z_t) b_{j,t}]^{y_{j,t}}. \tag{3}$$

Note that the first product above only needs to range from $t = 1$ until $\min(\tau, T_1(y))$ for the following reason. If event j cannot occur at time t given $H_{t-1} = h_{t-1}$ then $b_{j,t} = 0$. In this case then we always observe $y_{j,t} = 0$ and $\{\alpha_\theta(j, t | h_{t-1}, z_t) b_{j,t}\}^{y_{j,t}} = 1$. Also, $y_{j,t} = 0$ for all j with $t > T_1(y)$.

2.3 The likelihood function of the observed data

We derive the observed data likelihood function which is induced by the latent data pmf. The joint pmf at the observed data is

$$Pr_{\theta,\psi}\{G^1_{v^{obs}}(Y) = y^{obs}, G^2_c(Z) = z^{obs}, G^3_c(V) = v^{obs}\}. \tag{4}$$

Using the latent data joint probability mass function, (4) can be written as a sum over all realizations of the latent variables compatible with the observed

$$\sum_{\substack{v \in \mathbb{V}: \\ G^3_c(v) = v^{obs}}} \sum_{\substack{z \in \mathbb{Z}: \\ G^2_c(z) = z^{obs}}} \sum_{\substack{y \in \mathbb{Y}: \\ G^1_{v^{obs}}(y) = y^{obs}}} Pr_{\theta,\psi}(V = v, Z = z, Y = y). \tag{5}$$

$Pr_{\theta,\psi}(V = v, Y = y, Z = z)$ is proportional to $Pr_{\theta,\psi}(Y = y, Z = z)$ which is equal to

$$\prod_{t=1}^{\tau} Pr_{\theta,\psi}(Y_t, Z_t | Y_{[t-1]}, Z_{[t-1]}) = \prod_{t=1}^{\tau} Pr_{\theta,\psi}(Y_t, Z_t | H_{t-1}). \tag{6}$$

Therefore, (4) is proportional to

$$\sum_{\substack{y \in \mathbb{Y}: \\ G^1_{v^{obs}}(y) = y^{obs}}} \sum_{\substack{z \in \mathbb{Z}: \\ G^2_c(z) = z^{obs}}} Pr_{\theta,\psi}(Y = y, Z = z). \tag{7}$$

We now simplify (7) by summing the components of y and z after $d - \delta$. This is possible since the condition $G^1_{v^{obs}}(y) = y^{obs}$ only restricts the possible values of an arbitrary realization y of Y, up until $d - \delta$, i.e. it is equivalent with the condition $G^1_{v^{obs}}(y_{[d-\delta]}) = y^{obs}_{[d-\delta]}$. Similarly, $G^2_c(z) = z^{obs}$, is equivalent with $G^2_c(z_{[d-\delta]}) =$

$z^{obs}_{[d-\delta]}$. Because of this, the sum of all conditional probabilities of the components of y and z after $d-\delta$ given $y_{[d-\delta]}$ and $z_{[d-\delta]}$ must equal 1. Therefore, (7) is equal to

$$\sum_{\substack{y_{[d-\delta]}\in\mathbb{Y}_{[d-\delta]}:\\ G^1_{v^{obs}}(y_{[d-\delta]})=y^{obs}_{[d-\delta]}}} Pr_{\theta,\psi}(Y_{[d-\delta]}=y_{[d-\delta]}, Z_{[d-\delta]}=z^{obs}_{[d-\delta]}). \tag{8}$$

Repeating the arguments that lead to $Pr_{\theta,\psi}(Y=y,Z=z)\propto\prod_{t=1}^{\tau} Pr_\theta(Y_t=y_t|H_{t-1}=h_{t-1},Z_t=z_t)$ on the terms in above display, the joint probability mass of the observed data (4) seen to be proportional to what we define as the *ignorable likelihood contribution*, ignoring the coarsening

$$\begin{aligned} L^{ign}(\theta; y^{obs}, v^{obs}, z^{obs}, d, \delta) &:= \sum_{\substack{y_{[d-\delta]}\in\mathbb{Y}_{[d-\delta]}:\\ G^1_{v^{obs}}(y_{[d-\delta]})=y^{obs}_{[d-\delta]}}} \prod_{t=1}^{d-\delta}\prod_{j=0}^{n}\{\alpha_\theta(j,t|h(t-1),z_t)b_{j,t}\}^{y_{j,t}} \\ &= \sum_{\substack{y_{[d-\delta]}\in\mathbb{Y}_{[d-\delta]}:\\ G^1_{v^{obs}}(y_{[d-\delta]})=y^{obs}_{[d-\delta]}}} L^{latent}(\theta; y_{[d-\delta]}, z_{[d-\delta]}, d, \delta). \end{aligned} \tag{9}$$

For N independent subjects indexed by k the ignorable likelihood function is proportional to

$$L^{ign}(\theta; \{y^{obs,k}, v^{obs,k}, z^{obs,k}, d^k, \delta^k\}_{k=1}^N) = \prod_{k=1}^{N} L^{ign}(\theta; y^{obs,k}, v^{obs,k}, z^{obs,k}, d^k, \delta^k). \tag{10}$$

2.4 Partial derivatives of the ignorable log-likelihood

In the following, we compute the first and mixed second order partial derivatives of (10) with respect to θ. First, we simplify the notation. For a fixed realization $y_{[d-\delta]}\in\mathbb{Y}_{[d-\delta]}$ with $G^1_{v^{obs}}(y_{[d-\delta]})=y^{obs}_{[d-\delta]}$ we write y,z instead of $y_{[d-\delta]}, z_{[d-\delta]}$, and write $\sum_y L^{latent}(\theta;y)$ instead of the more cumbersome sum in the right hand side of (9). Clearly, $\sum_y L^{latent}(\theta;y)>0$. The log-likelihood function involves logarithms of sums over finite sets on the form

$$\log\{\sum_y L^{latent}(\theta;y)\}. \tag{11}$$

When there is no coarsening, the sum above consists of only one term and becomes $\log L^{latent}(\theta)$ which is the fully observed log-likelihood function.

2.4.1 Partial derivatives with only right censoring

We first compute the derivatives of the log-likelihood contribution in (10) in absence of coarsening. For each time t, then y_t is a column vector and P_t is a row vector. Define the local likelihood contribution as

$$L_t(\theta) = \prod_{j=0}^{n}\{\alpha_\theta(j,t|h_{t-1},z_t)b_{j,t}\}^{y_{j,t}}. \tag{12}$$

Note that exactly one factor is not equal to 1 since exactly one of the powers are 1 and the others are zero (say $y_f(t)=1$ for an index f and all other $y_{j,t}=0$ for $j\neq f$). Also, whenever $y_{j,t}=1$ then $b_{j,t}=1$, so $y_t=y_t\cdot b_t$. Therefore

$$\begin{aligned} \prod_{j=0}^{n}\{\alpha_\theta(j,t|h_{t-1},z_t)b_{j,t}\}^{y_{j,t}} &= \alpha_\theta(f,t|h_{t-1},z_t) \\ &= \sum_{j=0}^{n} y_{j,t}b_{j,t}\alpha_\theta(j,t|h_{t-1},z_t), \end{aligned} \tag{13}$$

so we may write

$$L_t(\theta) = \sum_{j=0}^{n} y_{j,t} b_{j,t} \alpha_\theta(j, t|h_{t-1}, z_t) = P_t^{b_t} y_t. \tag{14}$$

Its logarithm is

$$\log(L_t(\theta)) := \ell_t(\theta) = \log(P_t^{y_t}) = \log(P_t^{b_t}) y_t, \tag{15}$$

where the exponent and logarithm functions act element wise. The log-likelihood contribution is therefore

$$\ell(\theta) := \log\{L(\theta)\} = \sum_{t=1}^{\tau} \ell_t(\theta) = \sum_{t=1}^{\tau} \log(P_t^{b_t}) y_t. \tag{16}$$

Expressions for the partial derivatives, $\frac{\partial}{\partial\theta_j}\ell_t(\theta)$, and the entries of the Hessian, $\frac{\partial^2}{\partial\theta_l\partial\theta_j}\ell_t(\theta)$, are provided below.

2.5 Partial derivatives with general coarsening

With coarsening, the partial derivatives of (11) are

$$\frac{\partial}{\partial\theta_j} \log\{\sum_y L^{latent}(\theta; y)\} = \frac{\sum_y \frac{\partial}{\partial\theta_j} L^{latent}(\theta; y)}{\sum_y L^{latent}(\theta; y)}. \tag{17}$$

The mixed second order partial derivatives are

$$\frac{\{\sum_y \frac{\partial}{\partial\theta_l}\frac{\partial}{\partial\theta_j} L^{latent}(\theta; y)\}\{\sum L^{latent}(\theta; y)\} - \{\sum_y \frac{\partial}{\partial\theta_l} L^{latent}(\theta; y)\}\{\sum_y \frac{\partial}{\partial\theta_j} L^{latent}(\theta; y)\}}{\{\sum_y L^{latent}(\theta; y)\}^2}. \tag{18}$$

By the product rule, the derivatives can be expressed in terms of the time local latent likelihood $L_t^{latent}(\theta)$ contribution

$$\frac{\partial}{\partial\theta_j} L^{latent}(\theta) = L^{latent}(\theta) \sum_{t=1}^{\tau} \frac{\frac{\partial}{\partial\theta_j} L_t^{latent}(\theta)}{L_t^{latent}(\theta)}. \tag{19}$$

Expressions for the partial derivatives, $\frac{\partial}{\partial\theta_j} L_t^{latent}(\theta)$, and the entries of the Hessian,

$\frac{\partial}{\partial\theta_l}\frac{\partial}{\partial\theta_j} L^{latent}(\theta)$, are provided below.

2.6 Partial derivatives of related functions

In the following we compute all partial derivatives required to compute the gradients and Hessians of the log-likelihoods as described above. We first introduce some additional definitions and notation.

Let $1_{g\times h}$ indicate a matrix of $1's$ of dimension $g \times h$, and (a, A) be the concatenation of the number a onto the vector A. Let $\cdot$ indicate the element-wise product of two conformable matrices or vectors. Let $\exp(A)$ and $\log(A)$ for a matrix A, and powers A^a for two vectors of same length a, A, be the component wise exponential, logarithm, and powers, respectively.

Define $n_{nonabs} := n_{na} + 1$ to be the number of non-absorbing events including 0, and recall that $n = n_{na} + n_a$ is number of types of events except for the reference event. For a time t, let X_t $(1 \times k)$ be a design matrix including intercept and all covariates present in all linear predictors. It may contain arbitrary summaries of the history of the process up until time t and may be used to define the current state of the subject if that is meaningful. Recall that θ is a $(1 \times p)$ column vector. Define the linear parameter map $\phi := \phi(\theta)$ $(n \times k)$ that

maps the components of θ to where they are active in all n linear predictors η_j in the $(1 \times n)$ vector $\eta_t = (X_t \phi^T)$. Note that we allow linear predictors to share parameters, if desired.

In the following, let b be a realization of B_t^T, so, in contrast with the above, b_t has now dimension $1 \times n+1$. For a vector a, let a^- be a vector equal to a except that the first element has been omitted. Since the likelihood contribution is only evaluated at times when a subject is at risk, we always have $b_{0,t} = 1$ for $t \leq d - \delta$.

Let the denominator of the discrete hazard defined by the baseline category model be

$$Ref_t = 1 + \sum_{i=1}^{n} b_{i,t} \exp(\eta_i) = 1 + b_t^- \cdot \exp(\eta_t) 1_{n \times 1}. \tag{20}$$

Recall that for a given time t and data h_{t-1}, z_t, the jth discrete hazard function $P_{j,t}$ is identified with $\alpha_\theta(j, t|h_{t-1}, z_t) b_j(t)$, and the $1 \times n+1$ vector of probabilities P_t is

$$P_t = (1, b_t^- \cdot \exp(\eta_t))/Ref_t = b_t \cdot (1, \exp(\eta_t))/Ref_t. \tag{21}$$

Let $P_t^- = (b_t^- \cdot \exp(\eta_t))/Ref_t$.

2.6.1 The log-likelihood without coarsening

The partial derivatives of the log-likelihood $\frac{\partial}{\partial \theta_j} \ell_t(\theta)$ are given by

$$\frac{\partial}{\partial \theta_j} \ell_t = \frac{\partial}{\partial \theta_j} \log(P_t^{b_t}) y_t, \tag{22}$$

and the Hessian is the $p \times p$ matrix with entries on the form $\frac{\partial^2}{\partial \theta_l \partial \theta_j} \ell_t(\theta)$ with

$$\frac{\partial}{\partial \theta_l} \frac{\partial}{\partial \theta_j} \ell_t(\theta) = \frac{\partial}{\partial \theta_l} \frac{\partial}{\partial \theta_j} \log(P_t^{b_t}) y_t. \tag{23}$$

We now derive expressions for $\frac{\partial}{\partial \theta_j} \log(P_t^{b_t}) y_t$ and $\frac{\partial}{\partial \theta_l} \frac{\partial}{\partial \theta_j} \log(P_t^{b_t}) y_t$. Brackets after expressions indicate the dimension of the corresponding matrix.

The partial derivative of the linear map ϕ is

$$\phi_j' := \frac{\partial}{\partial \theta_j} \phi \quad (n \times k). \tag{24}$$

It is an indicator matrix with elements equal to 1 where the parameter θ_j is in the corresponding cell and else 0. Hence, the partial derivative of the linear predictors is

$$\frac{\partial}{\partial \theta_j} \eta_t = X_t \frac{\partial}{\partial \theta_j} \phi^T = X_t \phi_j'^T \quad (1 \times n), \tag{25}$$

Note that $\frac{\partial}{\partial \theta_j} \eta_t$ is just a constant so differentiating it again with respect to $\frac{\partial}{\partial \theta_l}$ gives the zero vector

$$\frac{\partial}{\partial \theta_l} \frac{\partial}{\partial \theta_j} \eta_t = \frac{\partial}{\partial \theta_l} X_t \phi_j'^T = 0 \quad (1 \times n). \tag{26}$$

Moreover,

$$\begin{aligned} \frac{\partial}{\partial \theta_j} \exp(\eta_t) &= (\frac{\partial}{\partial \theta_j} \eta_t) \cdot \exp(\eta_t) \\ &= X_t \phi_j'^T \cdot \exp(\eta_t) \quad (1 \times n), \end{aligned} \tag{27}$$

and the partial derivative of Ref_t is the scalar

$$\begin{aligned}\frac{\partial}{\partial\theta_j}Ref_t &= b_t^- \cdot \frac{\partial}{\partial\theta_j}\exp(\eta_t)1_{n\times 1}\\ &= b_t^- \cdot X_t\phi_j'^T \cdot \exp(\eta_t)1_{n\times 1}\\ &= X_t\phi_j'^T \cdot b_t^- \cdot \exp(\eta_t)1_{n\times 1}.\end{aligned} \tag{28}$$

To compute $\frac{\partial}{\partial\theta_j}\log(P_t^{b_t})$ we first compute the partial derivative of P_t, which is

$$\begin{aligned}\frac{\partial}{\partial\theta_j}P_t &= \left((0, b_t^- \cdot \frac{\partial}{\partial\theta_j}e^{\eta_t})Ref_t - (1, b_t^- \cdot e^{\eta_t})\frac{\partial}{\partial\theta_j}Ref_t\right)/Ref_t^2\\ &= (0, b_t^- \cdot X\phi_j'^T \cdot e^{\eta_t})/Ref_t - (1, b_t^- \cdot e^{\eta_t}) \cdot (b_t^- \cdot X_t\phi_j'^T \cdot \exp(\eta_t)1_{n\times n+1})/Ref_t^2\\ &= (0, X_t\phi_j'^T) \cdot P_t - P_t \cdot (X_t\phi_j'^T \cdot P_t^- 1_{n\times n+1})\\ &= P_t \cdot \big((0, X_t\phi_j'^T) - (X_t\phi_j'^T \cdot P_t^- 1_{n\times n+1})\big)\\ &= P_t \cdot A_t(j),\end{aligned} \tag{29}$$

where

$$A_t(j) := (0, X_t\phi_j'^T) - (X_t\phi_j'^T \cdot P_t^- 1_{n\times n+1}) \quad (1\times n+1). \tag{30}$$

Differentiation of each component $\log(P_{i,t}^{b_{i,t}})$ in $\log(P_t^{b_t})$ results in 0 when $b_{i,t} = 0$, and when $b_{i,t} = 1$ then $P_{i,t} > 0$ and

$$\begin{aligned}\frac{\partial}{\partial\theta_j}\log(P_{i,t}) &= \frac{1}{P_{i,t}}\frac{\partial}{\partial\theta_j}P_{i,t}\\ &= \frac{1}{P_{i,t}}P_{i,t} \cdot A_{i,t}(j)\\ &= A_{i,t}(j),\end{aligned} \tag{31}$$

therefore

$$\frac{\partial}{\partial\theta_j}\log(P_t^{b_t}) = A_t(j)b_t, \tag{32}$$

and the mixed second order partial derivative is

$$\frac{\partial}{\partial\theta_l}\frac{\partial}{\partial\theta_j}\log(P_t^{b_t}) = \frac{\partial}{\partial\theta_l}A_t(j)b_t, \tag{33}$$

where the partial derivative of $A_t(j)$ has the same dimension as $A_t(j)$ and is equal to

$$\begin{aligned}\frac{\partial}{\partial\theta_l}A_t(j) &= \frac{\partial}{\partial\theta_l}(0, X_t\phi_j'^T) - (X_t\phi_j'^T \cdot P_t^- 1_{n\times n+1})\\ &= -(X_t\phi_j'^T \cdot \frac{\partial}{\partial\theta_l}P_t^-)1_{n\times n+1},\end{aligned} \tag{34}$$

where the partial derivative of P_t^- is

$$\frac{\partial}{\partial\theta_j}P_t^- = P_t^- \cdot A_t^-(j), \tag{35}$$

with

$$A_t^-(j) := X_t\phi_j'^T - (X_t\phi_j'^T \cdot P_t^- 1_{n\times n}) \quad (1\times n), \tag{36}$$

and

$$\frac{\partial}{\partial\theta_l}A_t^-(j) = -(X_t\phi_j'^T \cdot \frac{\partial}{\partial\theta_l}P_t^-)1_{n\times n}. \tag{37}$$

2.6.2 The latent likelihood under coarsening

Recall that the partial derivative of the latent likelihood is

$$\frac{\partial}{\partial \theta_j} L^{latent}(\theta) = L^{latent}(\theta) \sum_{t=1}^{\tau} \frac{\frac{\partial}{\partial \theta_j} L_t^{latent}(\theta)}{L_t^{latent}(\theta)} \tag{38}$$

The partial derivative of the local likelihood contribution is

$$\frac{\partial}{\partial \theta_j} L_t^{latent}(\theta) = \frac{\partial}{\partial \theta_j} P(t) y_t, \tag{39}$$

where $\frac{\partial}{\partial \theta_j} P_t$ is given by (29). Dividing by $L_t^{latent}(\theta)$ we get

$$\begin{aligned} \frac{\frac{\partial}{\partial \theta_j} L_t^{latent}(\theta)}{L_t^{latent}(\theta)} &= \frac{\frac{\partial}{\partial \theta_j} P_t y_t}{P_t y_t} \\ &= A_t(j) y_t. \end{aligned} \tag{40}$$

The only remaining unknown term in (18) is the mixed term $\frac{\partial}{\partial \theta_l} \frac{\partial}{\partial \theta_j} L^{latent}(\theta)$, which is

$$\begin{aligned} \frac{\partial}{\partial \theta_l} \frac{\partial}{\partial \theta_j} L^{latent}(\theta) &= \frac{\partial}{\partial \theta_l} \{ L^{latent}(\theta) \sum_{t=1}^{\tau} A_t(j) y(t) \} \\ &= \{ \frac{\partial}{\partial \theta_l} L^{latent}(\theta) \} \sum_{t=1}^{\tau} A_t(j) y_t + L^{latent}(\theta) \sum_{t=1}^{\tau} \frac{\partial}{\partial \theta_l} A_t(j) y_t, \end{aligned} \tag{41}$$

were the partial derivatives are given by (19) and (34) correspondingly.

3 Appendix B: details on Monte Carlo Expectation Maximization

3.1 Shrinking support

We introduce some restrictions on the support of Y which will allow us to relatively easy construct a proposal pmf that incorporates information from the past and the future and guarantees that it will not propose realizations outside the support of the target pmf.

The support of Y_t given the past is assumed to shrink with time in the following sense. For each event j there is an index set $F_j \subset \{0, \dots, n\}$, such that F_j are the only events that have a possibility of occurring after event j. We assume that event 0 does not prevent any other event from occurring at a later time, so $F_0 = \{0, \dots, n\}$, and that the reference event 0 can always occur with positive probability given any past events prior to absorption, so $0 \in F_j$ for $j = 0, \dots, n_{na}$. Since nothing occurs after absorption, $F_j = \emptyset$ for $j = n_{na} + 1, \dots, n$.

For example, assume that there are four events: 0, 1, 2, 3, where 3 is absorbing and event 2 omits any further events of type 1. Therefore, $F_0 = \{0, 1, 2, 3\}$, $F_1 = \{0, 1, 2, 3\}$, $F_2 = \{0, 2, 3\}$, and $F_3 = \emptyset$. If events 1, 0 and 2 occur at times $1, 2$ and 3 then the support at time 4 is $\{0, 2, 3\}$. The coarsening is assumed to group nonabsorbing events with event 0. This implies that $V_{j,t} = 0$ for some $j \neq 0$ whenever event j is coarsened, and else $V_{j,t} = j$.

3.2 Generating compatible realizations

We explicitly define what a *compatible realization* means in the following. Note that a realization satisfies $y \in \mathbf{Y}$ if and only if $\sum_{j=0}^{n} b_{j,t} y_{j,t} = 1$ for each time t up until $\min(T_1(y), \tau)$. Let E_t be the event at time t with realization e_t and observed that the previous statement is equivalent with saying that for each time t with event e_t then e_t is compatible with the past $\prod_{1 \le s < t} I(e_t \in F_{e_s}) = 1$ and and future $\prod_{t < s \le \min(T_1(y), \tau)} I(e_s \in F_{e_t}) = 1$. Thus, given an arbitrary sequence of events up until some time u, one can check that it is compatible with the model if for each time t we have $\prod_{1 \le s < t} I(e_t \in F_{e_s}) \prod_{t < s \le u} I(e_s \in F_{e_t}) = 1$. When data is coarsened, an arbitrary sequence of events is therefore compatible with y^{obs} and the model if for each time t we have

$$\prod_{1 \le s < t} I(e_t \in F_{e_s}) I(e_t \in \{j : y_{j,t}^{obs} = 1\}) \prod_{t < s \le d - \delta} I(e_s \in F_{e_t}) = 1. \tag{42}$$

This allows us to generate events sequentially at times during which it is unknown exactly which event that occurred for the following reason. Say that we have generated a compatible sequence of events up until time $t-1$ or that all events are observed up until $t-1$, then we will have that $\prod_{1 \le s < t} I(e_t \in F_{e_s}) I(e_t \in \{j : y_{j,t}^{obs} = 1\}) = 1$ as long as $e_t \in \{j : y_{j,t}^{obs} = 1\}$. We cannot, however, always evaluate $I(e_s \in F_{e_t})$ for all $s > t$ since these events have not yet been sampled at times where it is unknown which event that occurred. This is not an issue as long as we can pick e_t such that there is at least one sequence of future events that can occur given the past and observed future. When e_s is observed then check if $e_s \in F_{e_t}$, and at times s when e_s is unknown, i.e. when $\sum_{j=0}^{n} y_{j,s}^{obs} > 1$, there is always a chance that event 0 occurred since $y_{0,s}^{obs} = 1$, since $0 \in F_j$ for $j = 0, \dots, n_{na}$, $F_0 = \{0, \dots, n\}$, and since an absorption has not yet occurred ($\sum_{j=na+1}^{n} y_{j,s}^{obs} = 0$). We are therefore certain that there is always some event future sequence of events $e_s \in F_{e_t}$ that are also compatible with the model and observed data, and it is enough to check $e_s \in F_{e_t}$ at times where e_s is observed.

The proposal is constructed as follows. Consider observed events e_t^{obs} that are equal to $*$ at times where it is unknown what event occurred ($\sum_{j=0}^{n} y_{j,t}^{obs} \geq 2$). Let J be an indicator matrix of same dimension as $y_{[d-\delta]}^{obs}$ with components $J_{j,t} = 1$ if event j can occur at time t given y_s^{obs} and v_s^{obs} for $s \geq t$, else 0, corresponding to the last two factors in (42). That is, for each $1 \leq t < d-\delta$ and $j = 0, \ldots, n$ set

$$J_{j,t} := y_{j,t}^{obs} \prod_{\substack{t<s\leq d-\delta: \\ e_s^{obs} \neq *}} I(e_s^{obs} \in F_j)\}, \tag{43}$$

and at $t = d-\delta$, set $J_t = y_t^{obs}$, since there is no future information available that can further restrict the set of possible events at time t.

3.3 Importance sampling

Sampling is performed sequentially from $t = 1, \ldots, d-\delta$ and at each t a sample y_t is generated. The conditional proposal pmf at time t given the past is set to $Q_{j,t} := \frac{J_{j,t}P_{j,t}}{\sum_{j=0}^{n} J_{j,t}P_{j,t}}$. Note that the support of Q_t is the set of events j such that $b_{j,t}J_{j,t} = 1$. Clearly, this set is always nonempty: event 0 is always in the support if the event is unknown at t ($e_t^{obs} = *$) since $J_{0,t} = 1$, and if the event is known then it is in the support since it must be compatible with the current observation and future observed events ($J_{e_t^{obs},t} = 1$), and with the past events. The proposal pmf of the entire event history is the product $Q(y) = \prod_{t=1}^{d-\delta} Q_t^T y_t$, where A^T indicates the transpose of the matrix A, and the likelihood of the sample is proportional to $Pr_\theta(Y_{[d-\delta]} = y_{[d-\delta]} | Z_{[d-\delta]}^{obs} = z_{[d-\delta]}^{obs}) = \prod_{t=1}^{d-\delta} P_t^T y_t$. Note that it is possible that Q_t places all probability mass at one event j, e.g. if $y_{j,t}^{obs} = 1$, in which case the sampled realization satisfies $y_{j,t} = 1$ and $y_{k,t} = 0$ for $k \neq j$. The sampling algorithm is summarized in Algorithm (1) in the main article, and is repeated m times before the normalized weights $\tilde{w}$ are computed for each individual.

3.3.1 The score and Hessian of $Q(\theta, \theta^r)$, and the observed information matrix

Recall that $\mathbf{Y}^{obs}$ is all the observed data for all subjects, and $\mathbf{Y}$ is the latent data for all subjects. Let the gradient and Hessian of the latent log-likelihood be

$$S(\mathbf{y}) = \frac{\partial \log f(\mathbf{y}|\theta)}{\partial \theta}, \quad H(\mathbf{y}) = \frac{\partial^2 \log f(\mathbf{y}|\theta)}{\partial \theta \partial \theta}, \tag{44}$$

Formulas for the gradient and Hessian of $Q(\theta, \theta^r)$ are given by [2]. The gradient is

$$\frac{\partial Q(\theta, \theta^r)}{\partial \theta} = E_{\theta^r}[S(\mathbf{Y})|\mathbf{Y}^{obs} = \mathbf{y}^{obs}], \tag{45}$$

and the Hessian is

$$\frac{\partial^2 Q(\theta, \theta^r)}{\partial \theta \partial \theta} = E_{\theta^r}[H(\mathbf{Y})|\mathbf{Y}^{obs} = \mathbf{y}^{obs}]. \tag{46}$$

Following [3] or [4], the observed information matrix used to compute standard errors is obtained at the last iteration and is equal to the negative of

$$\begin{aligned} \frac{\partial^2 \log f(\mathbf{y}, \theta^r)}{\partial \theta \partial \theta} =& E_{\theta^r}[H(\mathbf{Y})|\mathbf{Y}^{obs} = \mathbf{y}^{obs}] \\ &+ E_{\theta^r}[S(\mathbf{Y})S(\mathbf{Y})^T|\mathbf{Y}^{obs} = \mathbf{y}^{obs}] \\ &- E_{\theta^r}[S(\mathbf{Y})|\mathbf{Y}^{obs} = \mathbf{y}^{obs}]E_{\theta^r}[S(\mathbf{Y})|\mathbf{Y}^{obs} = \mathbf{y}^{obs}]^T. \end{aligned} \tag{47}$$

The score and Hessian of Q and the observed information matrix can all be approximated given Monte Carlo samples and importance weights, as discussed by [3]. Recall that k indicates a subject, j indicates a Monte Carlo sample, and in the following the indices i, l indicate components of the score and Hessian, e.g. $S_i(\mathbf{y})$ and $H_{i,l}(\mathbf{y})$. Let $\tilde{y}_{k,j}$ be sample j of subject k of the latent data generated by Algorithm (1), and $\bar{w}_{k,j}$ is the corresponding self-normalized weight, normalized across j for fixed k.

It follows that $\sum_{k,j} \bar{w}_{k,j} \log L^{latent}(\theta; \tilde{y}^{k,j}, z^k, d^k, \delta^k)$ is a Monte Carlo approximation of $Q(\theta|\theta^r)$ when Monte Carlo sampling has been performed with θ^r as parameter vector. Similarly, the expectation of the score in (45) can be approximated by

$$E_{\theta^r}[S_i(\mathbf{Y})|\mathbf{Y}^{obs} = \mathbf{y}^{obs}] \approx \sum_{k,j} \bar{w}_{k,j} S_i(\tilde{y}_{k,j}), \tag{48}$$

and the expectation in (46) and the first term in (47) can be approximated by

$$E_{\theta^r}[H_{i,l}(\mathbf{Y})|\mathbf{Y}^{obs} = \mathbf{y}^{obs}] \approx \sum_{k,j} \bar{w}_{k,j} \frac{\partial^2}{\partial\theta_i \partial\theta_l} \log f(\tilde{y}_{k,j}). \tag{49}$$

The second term in (47) is equal to

$$\begin{aligned} E_{\theta^r}[S_i(\mathbf{Y})S_l(\mathbf{Y})|\mathbf{Y}^{obs} = \mathbf{y}^{obs}] &= E_{\theta^r}[\sum_k S_i(Y_k) \sum_{k'} S_l(Y_{k'})|\mathbf{Y}^{obs} = \mathbf{y}^{obs}] \\ &= E_{\theta^r}[\sum_k \sum_{k'} S_i(Y_k) S_l(Y_{k'})|\mathbf{Y}^{obs} = \mathbf{y}^{obs}] \\ &= \sum_k E_{\theta^r}[S_i(Y_k)S_l(Y_k)|Y_k^{obs} = y_k^{obs}] \\ &+ \sum_{k \neq k'} E_{\theta^r}[S_i(Y_k)|Y_k^{obs} = y_k^{obs}] E_{\theta^r}[S_l(Y_{k'})|Y_{k'}^{obs} = y_k^{obs}], \end{aligned} \tag{50}$$

and the expectation of the products of score components in the end of (50) can be approximated by

$$\sum_k E_{\theta^r}[S_i(Y_k)S_l(Y_k)|Y_k^{obs} = y_k^{obs}] \approx \sum_{k,j} \bar{w}_{k,j} S_i(\tilde{y}_{k,j}) S_l(\tilde{y}_{k,j}), \tag{51}$$

while the second term in the end of (50) is a double sum of expectations each approximated by (48). The third term in (47) is approximately equal to zero at convergence.

3.3.2 Parameter specification and convergence criteria

Implementing the MCEM algorithm involves making choices such as the number of Monte Carlo samples at each iteration, parameter updating rules (e.g. step size), convergence criteria, and burn-in. There are many combinations of choices and alternative approaches that can be considered in future work to improve the performance of the method, compare with e.g. [5] and [6]. The following choices were made to keep the implementation simple while making the simulation studies in the main article feasible, and parameters were chosen based on a preliminary set of simulations.

The parameter were updated according to Algorithm (2) in the main article. First, the parameter sequence θ^r was initialized using EarlyCensor. The maximum number of iterations was set to 10. To stabilize the sequence of parameter updates, a cumulative weighted average of parameter estimates obtained from the MCEM procedure was generated, with weights defined proportionally to the number of Monte Carlo samples used in

the corresponding iteration relative to previous iterations. The number of Monte Carlo samples m_r was initially set to $m_1 = 10$ and increased by 5 with each iteration until $m = 25$ for all simulation studies except for model 1 scenario 2 where $m = 40$. See Supplementary Table 5 for a summary of the simulation settings. No burn-in and convergence criteria was used, and instead, the algorithm was allowed to run until the maximal number of iterations (10) was reached. Q was maximized iteratively based on the Monte Carlo estimates of the expected the gradient and Hessian of the latent likelihood.

4 Appendix C: additional simulation details and results

4.1 Data generating model parameters

Supplementary Table 2: True model parameters for the data generating model 1. States: S0 = not received GnRH or AA, S1 = received AA but not GnRH, S2 = received GnRH. S2 had S1 is an indicator variable equal to 1 only if the subject is in state S2 and has previously been in state S1, else 0.

Event	S0 intercept	S0 time	S1 intercept	S1 time	S2 intercept	S2 time	S2 had S1
AA	-0.772	-0.297	1.66	-0.021			
GnRH	0.621	-0.713	-1.62	-0.039	0.829	-0.023	0.191
Death	-4.66	-0.023	-3.382	0.003	-2.549	-0.007	0.511

Supplementary Table 3: True model parameters for the data generating model 2. States: S0 = alive and received no prior treatment, S1 = received treatment.

Event	S0 intercept	S0 time	S1 intercept	S1 time
Treatment	-5	0.05	-4	0.01
Death	-8	0.1	-10	0.2

Supplementary Table 4: True model parameters for the data generating model 3. States: S0 = alive and received no prior treatment, S1 = received treatment.

Event	S0 intercept	S0 time	S1 intercept	S1 time
Treatment	-4	0.4	0	-0.1
Death	-7	1	-8	0.9

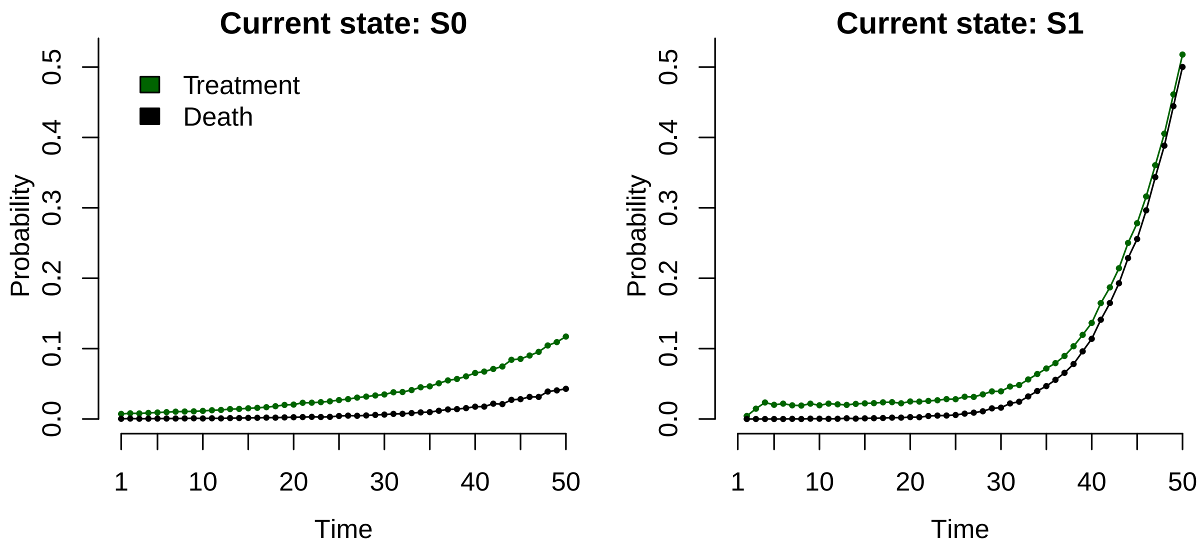

Figure 1: Approximate event probabilities (hazards) for data generating model 2 for times $1, \ldots, \tau = 50$, estimated via simulation of event histories of 100 000 subjects.

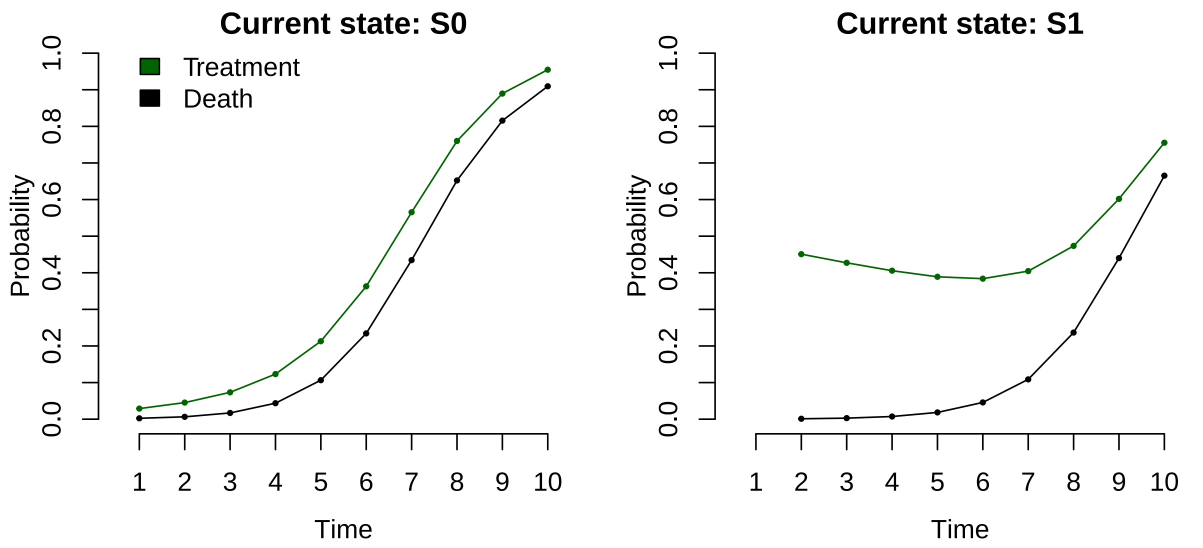

Figure 2: Event probabilities (hazards) for data generating model 3 for times $1, \ldots, \tau = 10$.

4.2 Simulation settings

Supplementary Table 5: Summary of simulation settings. n_{sim}=number of repetitions of the simulation, m=maximum number of Monte Carlo samples per individual for MCEM. Coarsened events always includes the event *Alive* (i.e., the reference event with index 0).

Model	Scenario	Events	Sample size N	n_{sim}	τ	Coarsening times	Coarsened events	m_{max}
			500	1024	10	$3-6$		25
1	1	AA, GnRH, Death	1000	1024	10	$3-6$	GnRH	25
			10000	256	10	$3-6$		25
			500	512	50	$5, 6, 20-29$		40
1	2	AA, GnRH, Death	1000	512	50	$5, 6, 20-29$	GnRH	40
			10000	128	50	$5, 6, 20-29$		25
			500	1024	10	$3-6$		25
1	3	AA, GnRH, Death	1000	1024	10	$3-6$	AA, GnRH	25
			10000	128	10	$3-6$		25
			500	512	50	$1-10$		25
2		Treatment, Death	1000	512	50	$1-10$	Treatment	25
			10000	128	50	$1-10$		25
			500	2048	10	$3-6$		25
3		Treatment, Death	1000	2048	10	$3-6$	Treatment	25
			10000	256	10	$3-6$		25

4.3 Results

Additional results are provided separately. Supplementary tables 6-20 listed below show bias, empirical standard error, and coverage, and supplementary Figure 3-7 listed below illustrate the same quantities.

List of supplementary tables

Supplementary Table 6. Model 1, scenario 1. Bias.

Supplementary Table 7. Model 1, scenario 1. EmpSE.

Supplementary Table 8. Model 1, scenario 1. Coverage.

Supplementary Table 9. Model 1, scenario 2. Bias.

Supplementary Table 10. Model 1, scenario 2. EmpSE.

Supplementary Table 11. Model 1, scenario 2. Coverage.

Supplementary Table 12. Model 1, scenario 3. Bias.

Supplementary Table 13. Model 1, scenario 3. EmpSE.

Supplementary Table 14. Model 1, scenario 3. Coverage.

Supplementary Table 15. Model 2. Bias.

Supplementary Table 16. Model 2. EmpSE.

Supplementary Table 17. Model 2. Coverage.

Supplementary Table 18. Model 3. Bias.

Supplementary Table 19. Model 3. EmpSE.

Supplementary Table 20. Model 3. Coverage.

List of supplementary figures

Supplementary figure 3. Model 1, scenario 1. Bias, EmpSE and coverage.

Supplementary figure 4. Model 1, scenario 2. Bias, EmpSE and coverage.

Supplementary figure 5. Model 1, scenario 3. Bias, EmpSE and coverage.

Supplementary figure 6. Model 2. Bias, EmpSE and coverage.

Supplementary figure 7. Model 3. Bias, EmpSE and coverage.

References

[1] Tsiatis A. Semiparametric theory and missing data. Springer Science & Business Media; 2007.

[2] Meilijson I. A fast improvement to the EM algorithm on its own terms. Journal of the Royal Statistical Society: Series B (Methodological). 1989;51(1):127-38.

[3] Robert C, Casella G. Monte Carlo statistical methods. Springer Science & Business Media; 2013.

[4] Louis TA. Finding the observed information matrix when using the EM algorithm. Journal of the Royal Statistical Society: Series B (Methodological). 1982;44(2):226-33.

[5] Celeux G, Chauveau D, Diebolt J. On stochastic versions of the EM algorithm [Ph.D. thesis]. INRIA; 1995.

[6] Caffo BS, Jank W, Jones GL. Ascent-based Monte Carlo expectation–maximization. Journal of the Royal Statistical Society: Series B (Statistical Methodology). 2005;67(2):235-51.
