# Supplementary figures and images for: Estimation in discrete time coarsened multivariate longitudinal models
Source: Stat Methods Med Res. 2023 Feb 12;32(4):806–19. doi: 10.1177/09622802231155010 (PMC10119900; doi:10.1177/09622802231155010)

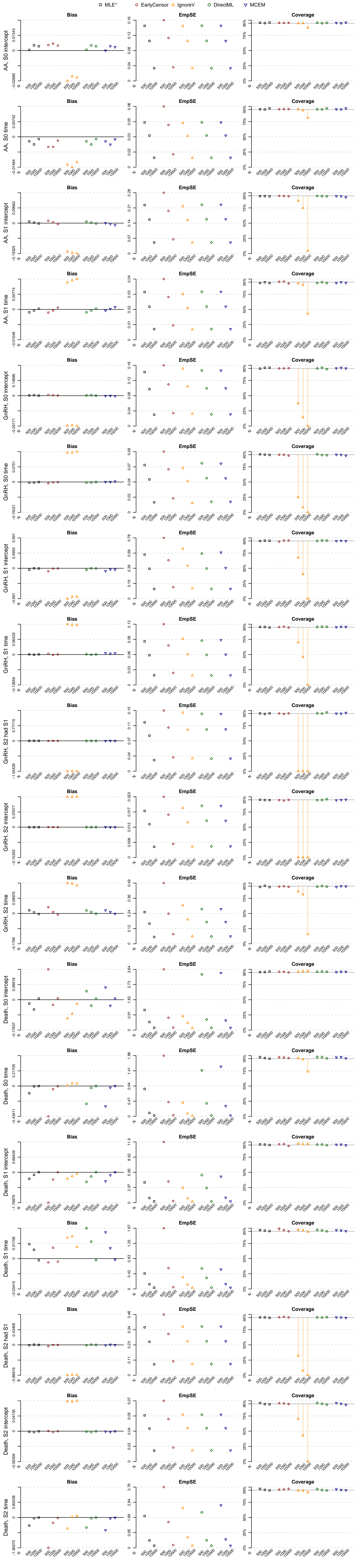

Supplement: sj-zip-3-smm-10.1177_09622802231155010 - Supplemental material for Estimation in discrete time coarsened multivariate longitudinal models [file sj-zip-3-smm-10.1177_09622802231155010.zip › SupplementaryFigures/SupplFig3.pdf]

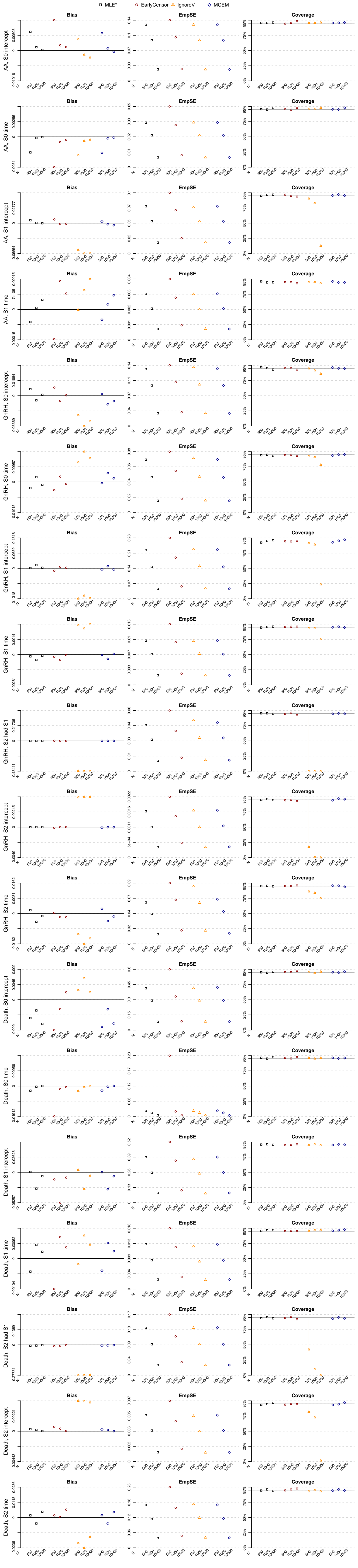

Supplement: sj-zip-3-smm-10.1177_09622802231155010 - Supplemental material for Estimation in discrete time coarsened multivariate longitudinal models [file sj-zip-3-smm-10.1177_09622802231155010.zip › SupplementaryFigures/SupplFig4.pdf]

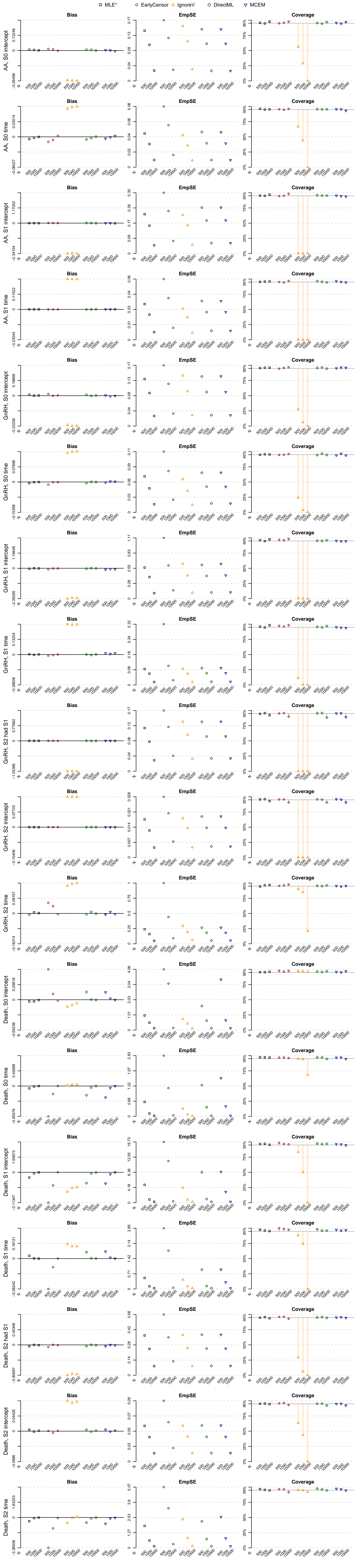

Supplement: sj-zip-3-smm-10.1177_09622802231155010 - Supplemental material for Estimation in discrete time coarsened multivariate longitudinal models [file sj-zip-3-smm-10.1177_09622802231155010.zip › SupplementaryFigures/SupplFig5.pdf]

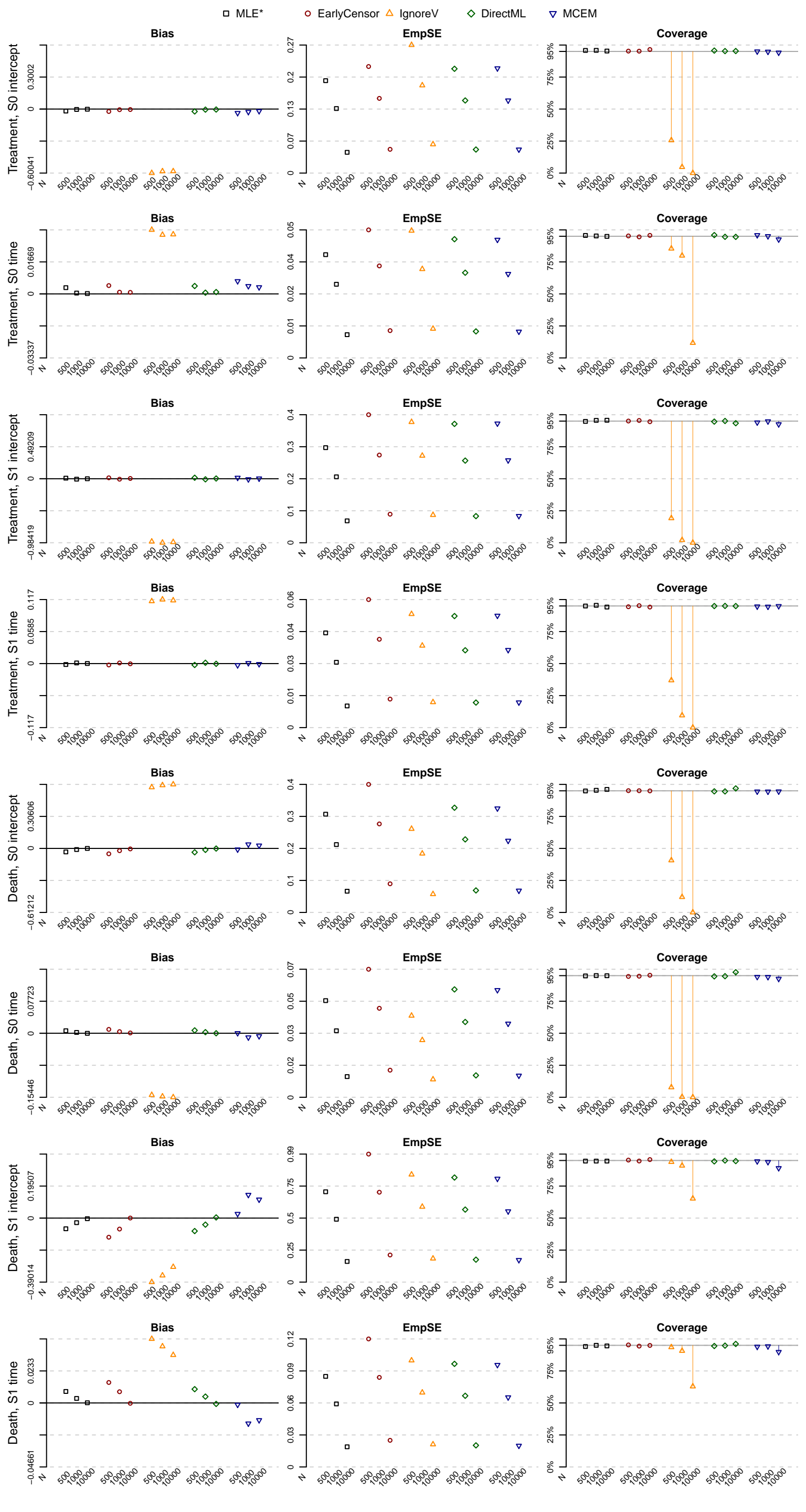

Supplement: sj-zip-3-smm-10.1177_09622802231155010 - Supplemental material for Estimation in discrete time coarsened multivariate longitudinal models [file sj-zip-3-smm-10.1177_09622802231155010.zip › SupplementaryFigures/SupplFig6.pdf]

□ MLE\*    ● EarlyCensor    ▲ IgnoreV    ◆ MCEM

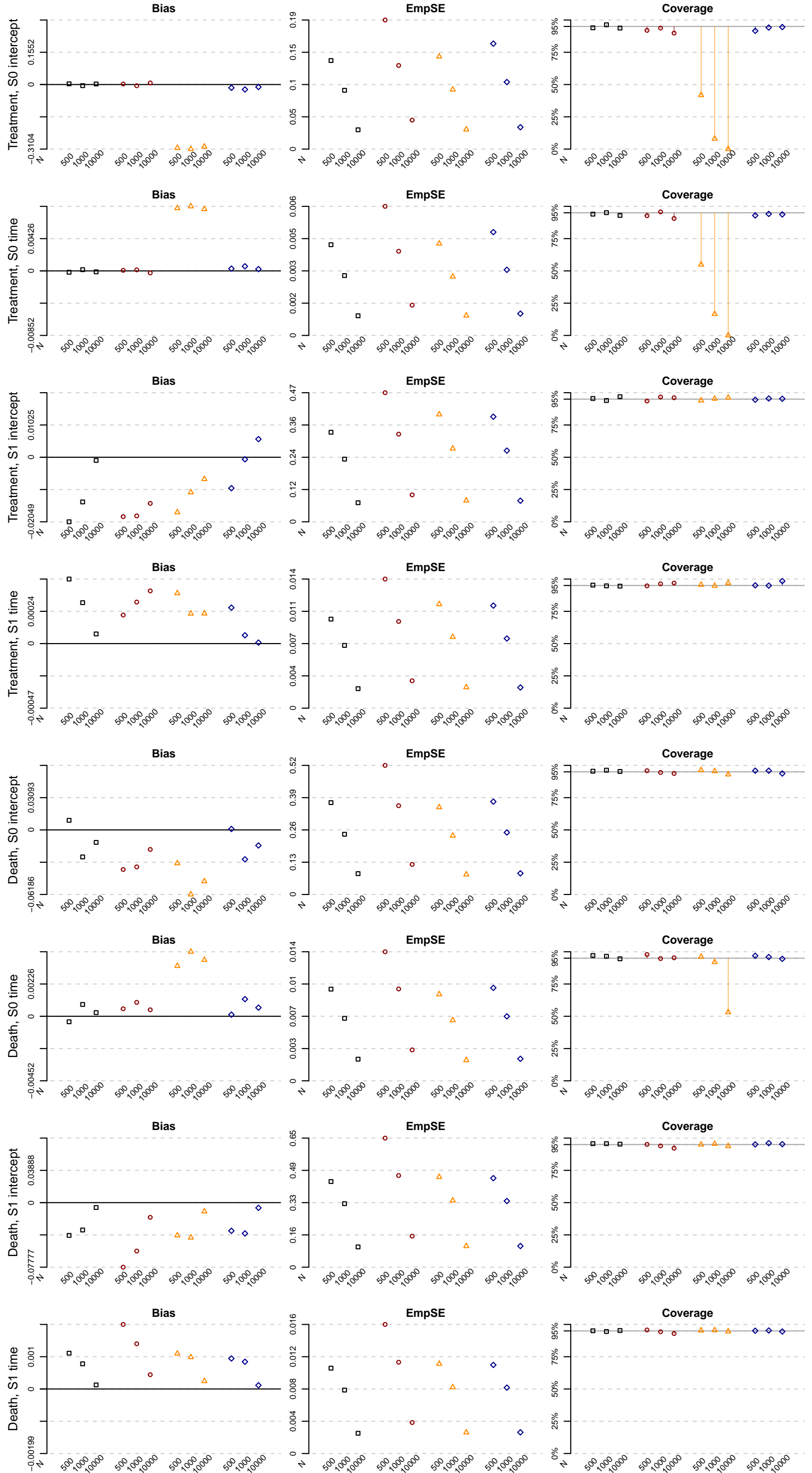

Supplement: sj-zip-3-smm-10.1177_09622802231155010 - Supplemental material for Estimation in discrete time coarsened multivariate longitudinal models [file sj-zip-3-smm-10.1177_09622802231155010.zip › SupplementaryFigures/SupplFig7.pdf]
